# A Comparative Pan-Genome Perspective of Niche-Adaptable Cell-Surface Protein Phenotypes in *Lactobacillus rhamnosus*

**DOI:** 10.1371/journal.pone.0102762

**Published:** 2014-07-17

**Authors:** Ravi Kant, Johanna Rintahaka, Xia Yu, Pia Sigvart-Mattila, Lars Paulin, Jukka-Pekka Mecklin, Maria Saarela, Airi Palva, Ingemar von Ossowski

**Affiliations:** 1 Department of Veterinary Biosciences, Faculty of Veterinary Medicine, University of Helsinki, Helsinki, Finland; 2 MTT Agrifood Research Finland, Jokioinen, Finland; 3 Institute of Biotechnology, University of Helsinki, Helsinki, Finland; 4 Department of Surgery, Jyväskylä Central Hospital and Institute of Clinical Medicine, University of Eastern Finland, Jyväskylä, Finland; 5 VTT Technical Research Center of Finland, Espoo, Finland; Wilfrid Laurier University, Canada

## Abstract

*Lactobacillus rhamnosus* is a ubiquitously adaptable Gram-positive bacterium and as a typical commensal can be recovered from various microbe-accessible bodily orifices and cavities. Then again, other isolates are food-borne, with some of these having been long associated with naturally fermented cheeses and yogurts. Additionally, because of perceived health benefits to humans and animals, numerous *L. rhamnosus* strains have been selected for use as so-called probiotics and are often taken in the form of dietary supplements and functional foods. At the genome level, it is anticipated that certain genetic variances will have provided the niche-related phenotypes that augment the flexible adaptiveness of this species, thus enabling its strains to grow and survive in their respective host environments. For this present study, we considered it functionally informative to examine and catalogue the genotype-phenotype variation existing at the cell surface between different *L. rhamnosus* strains, with the presumption that this might be relatable to habitat preferences and ecological adaptability. Here, we conducted a pan-genomic study involving 13 genomes from *L. rhamnosus* isolates with various origins. In using a benchmark strain (gut-adapted *L. rhamnosus* GG) for our pan-genome comparison, we had focused our efforts on a detailed examination and description of gene products for certain functionally relevant surface-exposed proteins, each of which in effect might also play a part in niche adaptability among the other strains. Perhaps most significantly of the surface protein loci we had analyzed, it would appear that the *spaCBA* operon (known to encode SpaCBA-called pili having a mucoadhesive phenotype) is a genomic rarity and an uncommon occurrence in *L. rhamnosus*. However, for any of the so-piliated *L. rhamnosus* strains, they will likely possess an increased niche-specific fitness, which functionally might presumably be manifested by a protracted transient colonization of the gut mucosa or some similar microhabitat.

## Introduction

Amongst the diverse bacterial communities (or so-called gut microbiota) that can flourish from the physicochemical and nutrient-rich conditions within the mammalian intestine are several commensal members of the Gram-positive lactic acid bacteria (LAB). Included here are a select few species of the *Lactobacillus* genus (e.g., *L. gasseri, L. reuteri*, *L. ruminis*, and *L. salivarius*) that appear recalcitrant to the washout conditions of the gastrointestinal (GI) tract, and thus able to coexist within the indigenous (or autochthonous) commensal population of gut microbes [1]–[3]. Although tending to be proportionally less numerous and distributed unevenly along the intestine [2], it is understood that for these gut-indigenous *Lactobacillus* species they will have acquired competitive genotypes that then allow them to be part of the intestinal microbiome encompassing a recognizable lifelong core of commensal bacteria. Rather significantly, much is speculated from a growing body of research that the autochthonous commensal microflora, via their composite physiological properties and intricate molecular relationships, have an inherent basal-level ability to impart certain metabolic, protective, and structural functions that help to preserve and promote a natural state of good intestinal health [4], [5].

Then again, many other lactobacilli are unable to occupy permanently a specific intestinal niche [1]–[3]. Instead, such bacteria tend to persist in the gut for a relatively short time and are ultimately lost in the fecal stream if not steadily replenished. While it is that a variety of species originating from the gut and other habitats (e.g., *L. acidophilus*, *L. casei*, *L. paracasei*, *L. plantarum*, *L. rhamnosus*, *L. fermentum*, *L. johnsonii*, *L. brevis*, and *L. delbrueckii*) tend to exemplify this transient pattern of intestinal colonization [2], several of their strains have gone on to be used as so-called probiotics [2], [6]. For this, each of these so-utilized *Lactobacillus* strains exerts a characteristic genomic bias for phenotypic traits that in the end presumably help maintain intestinal health and remedy a number of gut-origin health problems encountered by humans and animals [7], [8]. However, because most probiotic lactobacilli are viewed then as members of the allochthonous microbiota, they can only be contributory to the activities of a gut microbiome that is solely temporal in function.

Undoubtedly, it is through an evolution of favorable genomic adjustments that indigenous or transient gut-dwelling lactobacilli will have acquired the genes for different phenotypic traits enabling a particular niche specialization, and so allowing each their own respective adaptability for graded survival in the GI tract. Among the purported “niche factors” that are likely to have some potential in making functional contributions to mediating lactobacillar gut colonization are those with cell-surface phenotypes that help advance outward contact with the surrounding local environment. Likewise for the lactobacillar probiotics, it is generally presumed that the combined properties of a number of external cell wall structures are the requisite traits needed by these transient types when forming a physical interaction with host intestinal cells, and so then to convey any health benefits. Key in this regard, is a specific genomic profile that adds to the overall compositional complexity and diversity of the cell-surface architecture. Here, this is achieved largely by a variety of genome-encoded cell wall ligands, which, when expressed and assembled in place, offer a divergent and multifactorial mix of outer surface properties that not only promote intestinal adhesiveness and respond to changing conditions, but also help in defining the species−/strain-specific commensal-beneficial (probiotic) behavior of certain lactobacilli [9]. Thus far, some of the cell envelope components in such lactobacilli that have had already been identified and characterized include cell surface-associated/−anchored proteins (e.g., adhesins, pili, and S-layers), and as well certain cell wall carbohydrate polymers [10], [11]. As several of these surface structures are considered important factors for promoting adhesion to the intestinal mucosa and epithelium, many of the gut-transient *Lactobacillus* species have demonstrated a well-developed functioning propensity for re-establishing normalcy to microbial imbalances and for supporting protective activities, like pathogen antagonism and bacteria-host immune cell interplay [12], [13].

*L. rhamnosus* is a ubiquitous species with an ecological adaptiveness covering a range of bodily habitats, this being exemplified by the numerous different strains that can be isolated from the GI tract, respiratory airways, oral and vaginal cavities, lactating mammary glands, and clinical-type infections [14]–[16]. Other *L. rhamnosus* strains are typically associated with certain fermented milk products (e.g., cheese and yogurt) [17], but sometimes as well, they can be involved in beer spoilage [18]. Regarded as a natural inevitability, food-related isolates at some stage will also be found to exist in the gut environment. In point of fact, a few *L. rhamnosus* strains are some of the earliest colonizers of the human gut, although their presence can be linked to the period of infant breastfeeding [14], which then reflects a predisposition to be short-lived as microbiota members. Similarly, most *L. rhamnosus* isolates perceived as health-beneficial and thus developed as probiotics also share this short-term gut colonization pattern [19], [20]. Thus, by its own observed behavior, *L. rhamnosus* can be construed as a transient gut-dwelling species [2]. However, in this regard, standing apart somewhat from most strains is *L. rhamnosus* GG, an often-used human gut-isolated probiotic. Behaving slightly aberrantly, and in relative comparison, this strain is reported to outlast other gut *L. rhamnosus* strains owing to a genomic coding capacity that makes it, among other differences, extra persistent and better embedded along the intestinal muco-epithelium lining [20]. In such a context, it can be reasonably argued that the extent of *L. rhamnosus* GG longevity within the gut milieu, while not being viewed outright as autochthonous in nature, might instead be at least categorized as a less stringent form of allochthony [21].

*In silico* scrutiny of the recent-sequenced *L. rhamnosus* GG genome [20] has certainly proved useful in identifying the genetic basis for some of the cell-surface structures that are regarded responsible for the strong adhesion capacity and protracted gut persistence of this strain. Here, for instance, the proteinaceous surface architecture in *L. rhamnosus* GG cells that supports a network of mucoadhesive interactions has been characterized functionally to thus far involve at least three types of predicted sortase-anchored cell wall proteins. Included are the MBF (mucus-binding factor) [21] and MabA (modulator of adhesion and biofilm) [22] proteins as well as (and more predominantly) the assemblage of *spaCBA*-encoded pilin subunits, the so-called SpaCBA pilus [20], [23],[24]. More significantly here, given that these pili were shown recently to be collagen binding [25], it can be presumed that such an extracellular matrix (ECM)-binding capability will as well facilitate adhesion to a damaged or otherwise breached intestinal epithelium surface. Thus, by exhibiting these cell-surface protein phenotypes, *L. rhamnosus* GG cells can seemingly be well situated in the gut for a comparatively longer duration. This then strengthens the capacity of *L. rhamnosus* GG to confer some elements of basal protective immunity via varied host immune cell responses purportedly induced by some of its proteinaceous [23], [26]–[30] and carbohydrous [31], [32] cell envelope constituents. Inasmuch as having been supported by numerous studies involving *in vitro* and *in vivo* experimentation, for the *L. rhamnosus* GG strain, its diverse array of outer cell-surface characteristics are thought to have an essential utility in the physicochemical mechanisms underpinning the beneficial effects presumably associated with this transient gut-adapted commensal [33].

In addition to the *L. rhamnosus* GG genome, many others have now been sequenced for various different strains, including those isolated from habitats aside from the human gut. As of yet, the unexplored genomics of any niche-related advantages augmenting adaptability to particular conditions has only recently begun to be unveiled [34], [35]. Nonetheless, also to be found informative in this regard is to understand better what sort of possible genotype-driven phenotypic variation at the cell surface can exist amongst those *L. rhamnosus* strains with different or similar isolated origins. As one way to tackle this, and as well introduce a more specific ecological perspective of the genome, we aimed to conduct a pan-genomic study of *L. rhamnosus*. Here though, instead of making a typical broad functional survey, our intent was to use *L. rhamnosus* GG as a benchmark strain for genomic comparisons, but then place a focal emphasis on some of those encoded gene products that might presumably help shape the functionality of the outer surface proteinaceous character of other *L. rhamnosus* strains. Data obtained from a pan-genome, by definition the comprehensive genetic pool of both core and dispensable genes [36], [37], will help indicate whether a full gene repertoire encoding cell-surface phenotypes is determinable for the *L. rhamnosus* genome. Thus, in this study, we in effect sought to determine whether a narrow spectrum of genetic determinants contributes to the functional ecological diversity of the *L. rhamnosus* species and, with that, how it might be that certain cell surface-specific proteins are linked to host-niche specialization.

## Results and Discussion

### Phylogenomic reconstruction of *L. rhamnosus*

Often sometimes overlooked among the different bacterial strains being used to construct a pan-genome is an accounting of their exact or true isolated ecological origin. Here, the host source of the strain is an important influencing parameter when the pan-genome is interpreted and if not correct, makes any conclusions drawn less convincing or reliable. In this regard, reconstructing a genome-based phylogeny that would include our pan-genome strains can offer some added insight by showing possible correlations between the inferred phyletic relationships and any common origins. For instance, when isolates from similar habitats, but some of whose origins are clearly known, are seen to form a distinct genetic lineage, this might bring some level of reliability to those strains whose reported originating source is less certain.

**Figure 1:**
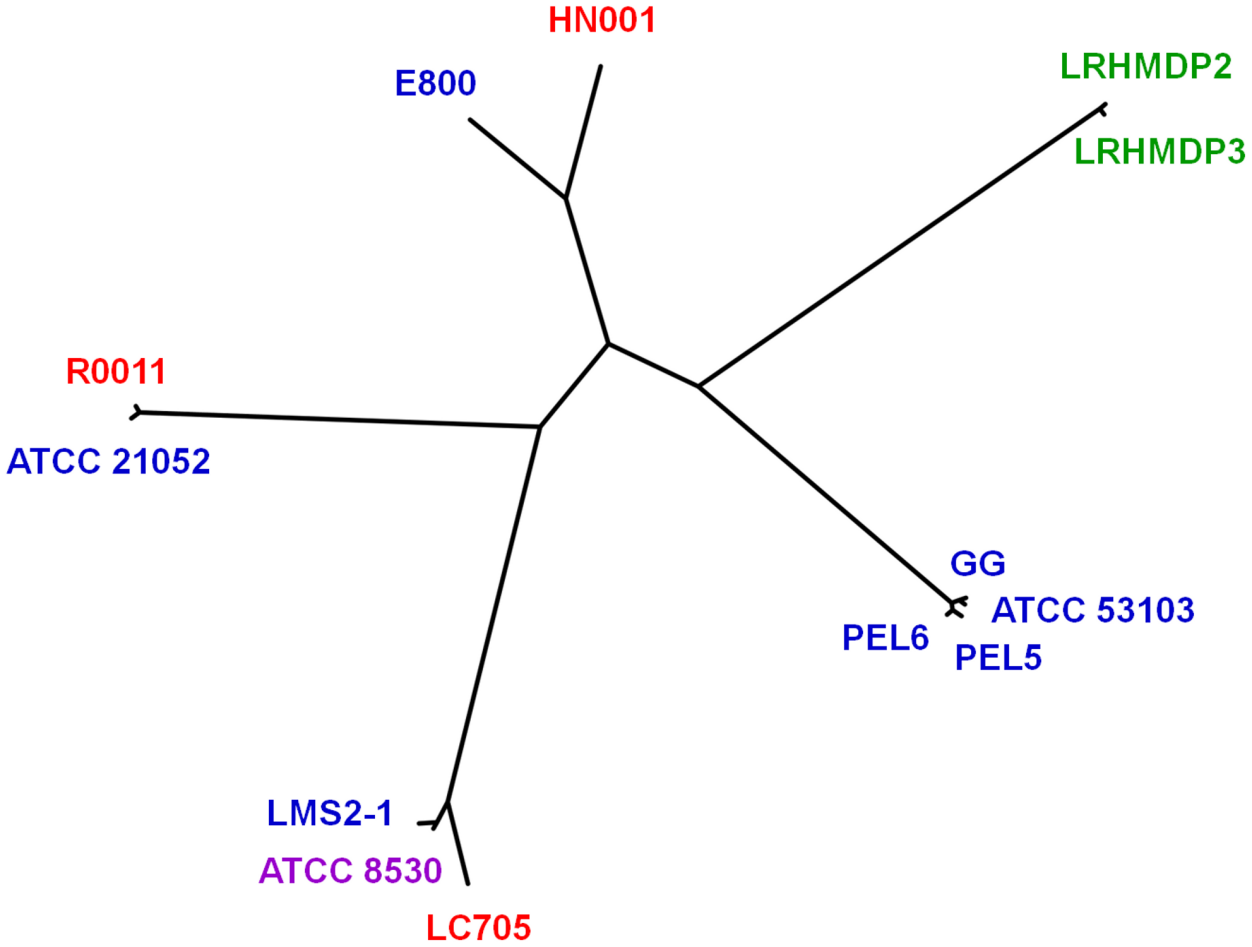
Phylogenomic tree of *L. rhamnosus*. For establishing the evolutionary relationships among the *L. rhamnosus* genomes, unrooted genome phylogenies based on aligned gene content were generated using the neighbor-joining method as described in Materials and Methods. Identities of the *L. rhamnosus* genomes (strains) are indicated. Origin and source of strains are grouped by color as follows: gut (blue), mouth (green), lungs (magenta), and dairy (red).

In our predicted pan-genome of *L. rhamnosus*, the strains involved come from natural habitats and origins, with these being the human gut and airways, dental infections and intestinal biopsies, and those that are milk-based (Table 1). However, because we calculated our pan-genome using a mix of both completed and draft *L. rhamnosus* genome sequences [20], [38]–[41], a cautionary caveat needs to be considered when interpreting the corresponding reconstructed phylogenomic tree (Figure 1). Still, even despite such limitations, but what might be expected, it would seem that among a few of the genomes there is an apparent clustering into clades based on the similar origins of the strains. For example, genomes of the two isolates from the human oral-dental cavity (LRHMDP2 and LRHMDP3) [38] are closely related and can pair together, while four genomes from strains with human gut-origins (GG, ATCC 53103, PEL5, and PEL6) branch out to generate a detached cluster.

**Table 1:** General attributes of the *L. rhamnosus* strains and genome sequences used in this study.

Strain	Source of Isolation	Genome Project Accession No.	Genome Status	Average Coverage	No. of Contigs	Genome Size (Mbps)*	G+C Content (%)	No. of ORFs**	Ref.
LC705	Milk	PRJNA59315	Complete	7x	1	3.03	46.6	3,033	[20]
ATCC 8530	Human airways	PRJNA162169	Complete	30x	1	2.96	46.8	2,977	[40]
GG	Human feces	PRJNA59313	Complete	11x	1	3.01	46.7	2,985	[20]
ATCC 53103	Human feces	PRJDA161983	Complete	9x	1	3.01	46.7	2,905	[39]
ATCC 21052	Human feces	PRJNA181588	Draft	149x	123	2.87	46.7	3,063	Unpub.^1^
HN001	Dairy starter	PRJNA55109	Draft	36x	94	2.91	46.6	2,864	Unpub.^1^
LMS2-1	Human gut	PRJNA55507	Draft	38x	162	3.11	46.5	3,209	Unpub.^1^
LRHMDP2	Infected dental pulp	PRJNA180948	Draft	17x	50	2.91	46.6	2,971	[38]
LRHMDP3	Infected dental pulp	PRJNA180949	Draft	17x	47	2.91	46.6	2,983	[38]
R0011	Cheddar cheese	PRJNA179908	Draft	32x	10	2.90	46.7	2,782	[41]
E800	Human feces	PRJNA236793	Draft	20x	83	3.03	46.6	3,107	This study
PEL5	Human gut biopsy	PRJNA237053	Draft	20x	81	2.99	46.7	2,922	This study
PEL6	Human gut biopsy	PRJNA237054	Draft	20x	71	2.88	46.7	2,899	This study

*Average genome size is 2.96 Mbps.

**Average number of ORFs is 2,977.

1 Unpublished.

On the other hand, it is all too apparent that the genomes from *L. rhamnosus* strains with dairy-sourced origins (R0011, HN001, and LC705; see Table 1) do not cluster together according to shared genotypes that are related to milk-based environments. Instead, each of these genomes forms a separate branch and is phyletically linked to the genome from a human (gut and/or lung) isolate (Figure 1). Given this genomic context, and with none of the dairy strains showing an overly strong genomic relatedness to one another, this might then suggest that their so-reported isolated-origin does not reflect the actual source where they had first originated, and alternatively can be human-related. Then again, but also speculatively, another interpretation is worth considering. For instance, since the genomes of the intestinal isolates (LMS2-1, E800, and ATCC 21052) that form a separate branch with one of the genomes from the dairy strains are noticeably absent from the “gut-origin” clade mentioned above, this could also mean these three strains are merely in-transit gut-isolates that had actually first originated elsewhere (e.g., food, vegetation, or soil). Nonetheless, although each of these interpretations is arguably plausible, to prove either one or the other would likely necessitate the inclusion of additional genome sequences in a wider phylogenetic reconstruction of *L. rhamnosus*. However, while it is so-perceived that reconstructed phylogenomic lineages can help distinguish some *L. rhamnosus* strains according to their isolation source, and thusly their ensuing adaptation to close-related habitats, it is also reasonable that different aspects of the evolutionary and ecological relationships among the other strains will still be inferable from each genome sequence within the context of our pan-genome.

### *L. rhamnosus* genome sequences and general features

To build the multiple genomic alignments needed for generating a pan-genome, 13 complete or draft genome sequences from *L. rhamnosus* strains representative of isolates from a varied set of natural habitats (see above) were obtained either by our high throughput sequencing (*n* = 3) or through availability in public databases (*n* = 10) as of June 2013. For annotating the three new draft genomes (from strains PEL5, PEL6, and E800), each of the assembled sequences was run on an automatic annotation pipeline, with the results undergoing some manual curation afterward (see Materials and Methods for more details.) A complete listing of gene annotations for these new sequenced genomes is provided as supporting information (see in Table S1). DNA sequence data for each of the genomes was deposited in GenBank under the accession numbers JDFQ00000000 (PEL5), JDFR00000000 (PEL6), and JDRW00000000 (E800). Of note, while some individual sequences from a few of the strains are representative of plasmids, our study did not include further examination of this genre of extrachromosomal DNA.

The general features of the three new *L. rhamnosus* genome sequences, as well as those of 10 reasonably good-quality sequenced genomes that we had retrieved from the NCBI RefSeq database, are compiled and presented in Table 1. It should be mentioned that two of the genomes do in fact come from different isolates of the same strain (i.e., *L. rhamnosus* GG or ATCC 53103), but whose sequences different research groups determined independently [20], [39]. Nonetheless, with a mix of different DNA sequencing platforms having had been used, the average coverage of assembled genome sequences is between 7-fold (LC705) and 149-fold (ATCC 21052). The number of contigs range from one, as with those genomes that were completely sequenced (LC705, GG, ATCC 8530, and ATCC 53103), to as many as 162, as we find with the draft genome sequence from strain LMS2-1. The range of sizes (based on the total contig lengths) among the 13 genomes is not too wide, covering 2.87 to 3.11 Mbps (ATCC 21052 and LMS2-1, respectively), and with 2.96 Mbps being the overall average. The G+C content of the 13 genomes shows only minor differences from one another, varying from 46.5 to 46.8%. The numbers of predicted protein-encoding ORFs in these genomes are between 2,782 and 3,209, which is then equivalent to an overall 14.2% difference.

### *L. rhamnosus* pan-genome

**Figure 2:**
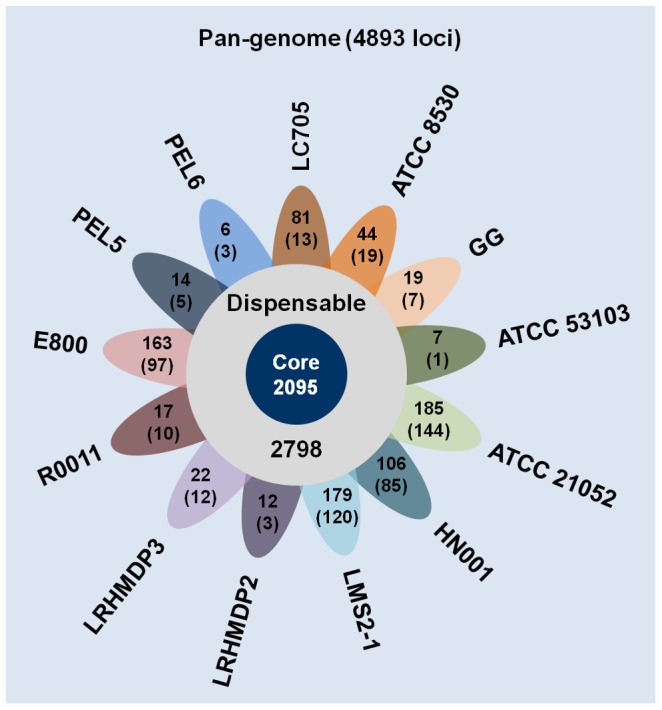
The pan-genome of *L. rhamnosus*. A flower-plot schematic representation illustrates the number of predicted core (2,095) and dispensable (2,798) genes that together make up the *L. rhamnosus* pan-genome (4,893 loci). Shown in the flower petals are the numbers of loci per genome that are predicted to be either unique or ORFan-like (parenthesized). Names of the *L. rhamnosus* genomes (strains) are indicated. All annotated genes are listed in Table S1.

**Figure 3:**
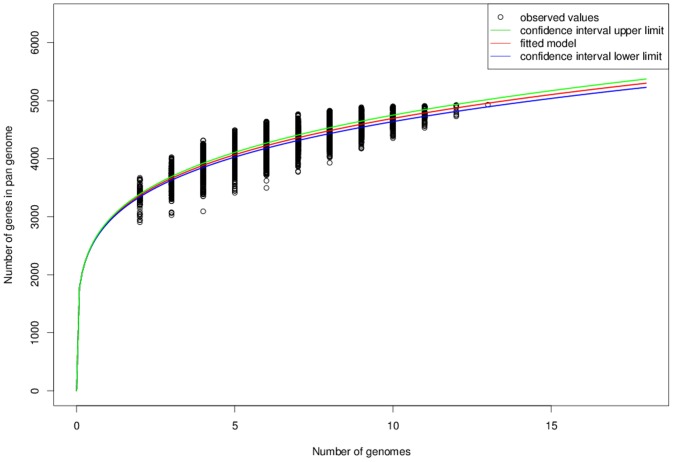
The pan-genome development plot of *L. rhamnosus*. Shown is the progression of the *L. rhamnosus* pan-genome as additional strain-genomes are included. Pan-genome development was calculated with R statistical programming language and using Heap’s Law (see Materials and Methods).

To gain an overall approximation of the total gene pool for *L. rhamnosus,* we had calculated the pan-genome based on the 13 genomes (see above) using the score ratio value (SRV) method together with the EDGAR software platform (see Materials and Methods for further details). Results obtained from these calculations suggest that the size of the *L. rhamnosus* pan-genome can be estimated to include 4,893 genes (Figure 2 and Table S1), which is only 1.6-fold the size of the average gene number (2,977) calculated from the 13 genomes. By comparison, the number of genes in the pan-genome of the taxonomically related *L. casei* species is about 3.2 times more than that averaged from its strain-genomes [42]. Here, it would appear that a higher level of genome instability is apparent for *L. casei* and more so than with the *L. rhamnosus* species, whose genome has been reported to encode fewer transposases [35], and which in itself might contribute to it being relatively more stable. Nonetheless, based on the predicted size (and inferred content) of the pan-genome, it is clear that evolutionary-driven genome instability and plasticity in *L. rhamnosus* is providing sufficient genotype-phenotype variability, and thus what supports the flexible capacity of this species to adapt to various ecological niches and environments. Moreover, when plotted data of the number of genes in the pan-genome as a function of the number of sequenced genomes (Figure 3) are fitted according to Heap’s Law, this then gives α = 0.79, and is suggestive of an open pan-genome [36], [37]. As such, this would be consistent with the *L. rhamnosus* species having multiple habitats and a projected propensity to undergo lateral gene transfer (LGT). However, even though this pan-genome was created with what we consider are good-quality genome sequences, it should be noted that since a sizable portion of the pan-genome includes several draft genomes, it could be anticipated that a greater number of fragmented genes will be identified and annotated. Consequently, this might then lead to an overestimation of predicted loci, thus artificially inflating the total size of the pan-genome.

Upon closer inspection of the pan-genome development plot (Figure 3), and if one follows the upper observed values, the curve seems to plateau at about just below 5,000 genes. It is perceptible here that with each additional genome beyond the tenth genome, fewer new genes are being identified and thus there begins to be a less appreciable increase in the pan-genome size. This is in fact also supported by the core genome development plot (data not shown), which shows a noticeable reduction of the core genome with the inclusion of every added genome sequence. Added to this, the pan-genome data of the taxonomically close *L. paracasei* species was interpreted similarly [43]. Following such a trend, one might predict that the *L. rhamnosus* pan-genome would then likely exhibit an eventual progression to a more closed status. As such, it is plausible that only a few more strains representing a mix of different isolated-origins would be all that is needed for building a pan-genome that can sufficiently approximate the totality of genome variability based on acquired new gene content. However, even with the possible prospect of a less expanding pan-genome, *L. rhamnosus* genomes are still to be viewed as dynamically evolving entities. Here, it is then to be expected that any continued genetic upheaval will be less an outcome of individual gene loss or gain, but will rather instead rely on other variable influences such as single-nucleotide polymorphic changes, indel-related alterations, or the sometimes presence of extrachromosomal mobile elements. Such a genome scenario would continue to guarantee the *L. rhamnosus* species has enough genetic possibilities to further build up on its already established adaptive resilience and thus evolve those modified phenotypes needed for surviving in any other promising niche habitats that should become accessibly available.

### *L. rhamnosus* core genome

Among the ORFs in the pan-genome are those that constitute the core genome for a particular species. By definition, the core genome includes those orthologous genes with predicted functions that are recognized essential to cell viability and thus common to all strains [36], [37]. For the *L. rhamnosus* core genome, individual ORF identities were determined using the methodology as described in Materials and Methods. The size of the core genome is estimated at around 2,095 genes and accounts for about 43% of the entire pan-genome (Figure 2). Incidentally, the portion of the core genome encompassing the *L. paracasei* pan-genome is also 43% (i.e., 1,800 out of 4,200 genes) [43], whereas for the *L. casei* pan-genome it is only 29% (i.e., 1,715 out of 5,935 genes) [42]. Inferred from these percentages, it would seem that *L. rhamnosus* and *L. paracasei* have similar amounts of genetic variation, but it appears that despite being a taxonomic cousin, *L. casei* is comparatively much more diverse genomically than the other two species.

Additional details about the *L. rhamnosus* core genes, including their annotations, can be found in Table S1. Here, many of the different loci involved in essential housekeeping functions (e.g., DNA replication, mRNA transcription, protein synthesis and degradation, carbohydrate transport and biosynthesis/utilization, and cellular adhesion) are expectedly present, with some genes likely also grouped into operon-like islands. The overall genetic composition of the *L. rhamnosus* core genome tends to generally mirror that found for the other two members of the “casei” group of lactobacilli [42], [43], albeit with characteristic inbuilt “species-specific” differences, including some already identified from an earlier comparative genomics analysis of *L. rhamnosus* [34]. However, it is worth mentioning that included among the *L. rhamnosus* core genome are at least 75 genes that cannot be found in any other *Lactobacillus* species whose complete or good-quality draft genome sequences have so far been deposited in the NCBI RefSeq database (up to June 2013) (Table S1). As would appear somewhat expected of this type of broad comparison of genomes, the majority of these loci are annotated as hypothetical proteins, and with most of the other ORFs specifying predicted proteins that have functions as membrane transporters, transcriptional regulators, and glycosyltransferases.

### *L. rhamnosus* dispensable genome

The remaining part of the *L. rhamnosus* pan-genome, typically referred to as the dispensable (or accessory) genome, is then what actually defines the diversity of this species. Characteristically, the dispensable genome contains ORFs whose presumed functions might be deemed nonessential, but otherwise can still offer a selective-to-competitive advantage to the different strains [36], [37]. For *L. rhamnosus*, these so-classified accessory genes are the ones shared by at least two but not all genomes in the pan-genome, and are here estimated to include about 2,798 loci (Figure 2). Included in the dispensable genome is a set of unique (or strain-specific) genes that can only be found in each individual *L. rhamnosus* strain-genome. The numbers of these genes per each genome are indicated in Figure 2. In total, there are roughly 855 unique genes, with these comprising around 30% of the dispensable genome. Also shown in Figure 2 are the per-genome numbers of ORFan-like sequences, which we define in this study as those genes in the various *L. rhamnosus* strains that show no homologous match to any other published or database-deposited *Lactobacillus* genomes. These total 519 in number and make up about 18% of the dispensable genome.

Overwhelmingly, most of the accessory genes in the *L. rhamnosus* pan-genome are annotated as hypothetical proteins or proteins with an unknown function (Table S1). As such, these types of gene annotations no doubt reflect a “black-box” of sorts. Likewise, it generally remains uncertain what proportion of so-annotated ORFs would actually encode functional protein that is then produced in cells. Moreover, while it is credible to accept that a disproportionate number of such gene annotations can be found in those *L. rhamnosus* genomes that are sequenced completely, for the draft genomes some of the genes specifying hypothetical or unknown-function proteins might be representing fragmented ORF sequences, which would then cause their numbers to be inflated and overrepresented. Irrespective of this possibility, among the annotated ORFs with recognized functions and phenotypes, some are related to extrachromosomal mobile elements, while a collection of others are those that have predicted roles in membrane transport, transcriptional regulation, carbohydrate biosynthesis and utilization, and cell-surface adhesion. Taken together, these results for *L. rhamnosus* are not entirely too surprising as earlier pan-genome studies involving two taxonomic relatives (i.e., *L. casei* [42] and *L. paracasei* [43]) have reported a reasonably similar gene profile for their own dispensable genomes.

### *L. rhamnosus* pan-secretome

Because the main intent of this study is to provide a closer genomic look at some of the proteinaceous cell-surface phenotypes of the *L. rhamnosus* species, we also decided to include a pan-secretomic survey of the 13 genomes. For this, we directed our analysis toward those putative classical secretory proteins and/or Sec-pathway exported proteins being encoded in the *L. rhamnosus* genomes. *In silico* identification of these secretome proteins was based on Gram-positive SignalP 4.1 predictions of the strain-genomes. Additional details about the methodology used are described in Materials and Methods.

**Figure 4:**
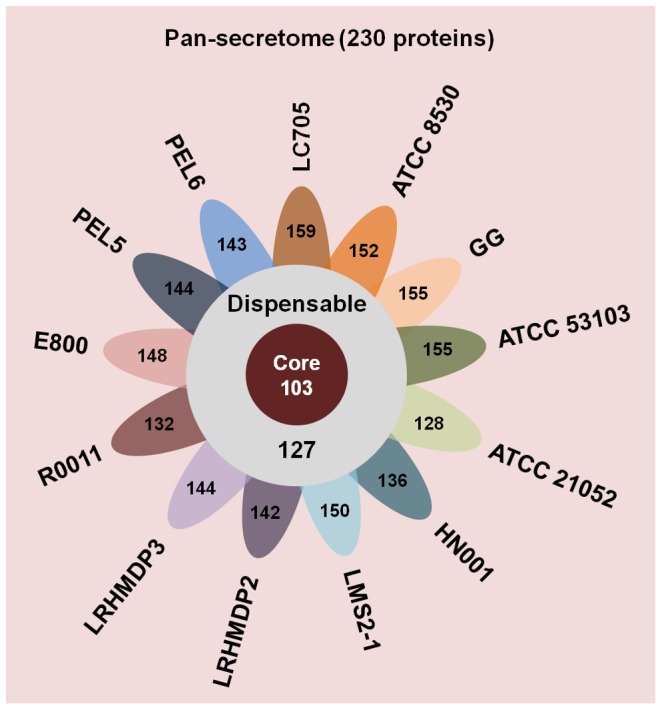
The “classical” pan-secretome of *L. rhamnosus*. A flower-plot schematic representation depicts the main components of the SignalP-predicted *L. rhamnosus* pan-secretome (230 proteins). Shown are the number of core (103) and dispensable (127) proteins and the total numbers of classically secreted proteins per each genome (flower petals). Names of the *L. rhamnosus* genomes (strains) are indicated. All annotated secreted proteins are listed in Table S2.

The predicted size of the *L. rhamnosus* “classical” pan-secretome is calculated at approximately 230 proteins (Figure 4 and Table S2), which is then equivalent to only 4.7% of the full pan-genome. The pan-secretome is itself made up of about 103 core proteins, with the remainder encompassing 127 dispensable proteins (Figure 4 and Table S2). The total numbers of SignalP-predicted secreted proteins per each genome are also calculated (Figure 4). Among the annotated loci in the predicted core secretome (Table S2), there are expectedly several for an assortment of different ABC transporter/substrate-binding proteins and various types of sortase-dependent LPXTG-motif proteins, including some putative adhesins. Moreover, there are at least a dozen genes annotated as hypothetical proteins. Also included in the core secretome are a number of those surface proteins that had been already characterized functionally in the *L. rhamnosus* GG strain (see following section below). On the other hand, and what is more difficult to interpret, an overwhelming number (∼75) of the annotated proteins in the so-deduced dispensable secretome (Table S2) are categorized as hypothetical. To a reduced extent than with the core secretome, but still found similarly, most of the other “dispensable proteins” had predicted functions as membrane-bound transporters and LPXTG-like surface adhesins. Interestingly though, among the latter annotated types are those predicted genes for the SpaCBA pilus-related proteins (see following section below).

Of some additional interest, and amongst the secretome proteins having been identified, there was a collection of proteins whose C-terminal contains the so-defined WxL domain. In an earlier study [35], it was reported that the *L. rhamnosus* ATCC 53103 genome encodes three separate gene clusters for this type of surface protein, i.e., LRHM_0182 to LRHM_0184, LRHM_0555 to LRHM_0564, and LRHM_1698 to LRHM_1704. Loci for two of these clustered DNA regions (LRHM_0182 to LRHM_0184, and LRHM_1698 to LRHM_1704) were seen to be part of the core genome, as homolog counterparts could be found in all 13 genomes of the pan-genome (Table S1). As might be expected, some of these proteins were also detected as being part of the predicted core secretome (Table S2), except for the LRHM_1702 homolog, which could not be identified by SignalP predictions. For the LRHM_0555 to LRHM_0564 genomic region, because a few of the loci were missing from four of the genomes in the pan-genome (i.e., ATCC 21052, E800, HN001, and R001), certain genes that correspond to this WxL domain-related clustering were then part of either the core or dispensable genomes (Table S1). Likewise, a similar portioning of these genes (proteins) was observed in the core and dispensable secretomes (Table S2).

It is noteworthy to mention that whereas a wide cross-section of classically secreted adhesive-like surface proteins could be identified in this pan-secretomic profiling of *L. rhamnosus*, numerous non-classically secreted adhesins were then to be excluded. One of these of particular interest is a gene annotated as a collagen-binding protein, which itself can be found only in the genome of the E800 strain (E800_2250), and so judged to be one of the unique (strain-specific) loci of the *L. rhamnosus* dispensable genome. SignalP predictions of the pan-genome ORFs did not identify this 379-residue protein, as it would appear to lack a classical N-terminal signal peptide sequence. Moreover, based on primary structure predictions using the Secretome 2.0 and TatP 1.0 programs (data not shown), it is then more likely to be one of the non-classically secreted proteins in the E800 strain. Incidentally, a BlastP search of the NCBI database revealed that the most-related homologs are encoded in the plasmid genome of the LC705 strain (pLC705_00003), but also in the genome of various *L. casei* and *L. paracasei* strains, and as well in *Enterococcus faecium*, a potential gut pathobiont (data not shown). Interestingly, the E800 strain has been shown to adhere strongly to mucosal substrates much like the GG strain [44], but to our knowledge, no reported study has yet established its ability to bind collagen. The other *L. rhamnosus* strains (e.g., *L. rhamnosus* GG) having been characterized previously as such, but lacking this particular collagen adhesion gene, must instead presumably rely on other types of collagen-specific adhesins [25]. However, consistent with a conceivable localization at the cell surface, this particularly unique collagen-binding protein might offer some competitive advantage to the E800 strain when colonizing epithelium-rich surroundings.

### Cell-surface protein phenotype variation in *L. rhamnosus* strains

For this part of the study, our examination is focused on the potential genomic variation of those loci encoding the membrane- and cell wall-associated proteins, some of which are identified from our pan-secretomic survey, but more precisely, those that have been characterized previously at a molecular and biochemical level in *L. rhamnosus* GG or other related species. Our goal here is to pinpoint which of the recognized-to-be-functional outer surface proteins can offer a selective or possibly competitive advantage to different strains, and thus facilitate part of their respective adaptation to a specific niche environment. Accordingly, included are gene products that have a postulated or confirmed role in cell-surface adhesiveness, and which are to some extent, presumably or otherwise involved in bacteria-host immune cell interactions.

#### SpaCBA and SpaFED piliation

Previously, comparative sequence analysis of the *L. rhamnosus* GG genome had revealed the occurrence of two different pilus-encoding islands that have come to be known as the *spaCBA* and *spaFED* operons [20]. Each of the pilus operons is comprised of three pilin subunit genes and one pilin-specific sortase gene, with these being LGG_00444 (*spaC*), LGG_00443 (*spaB*), LGG_00442 (*spaA*), and LGG_00441 (*srtC1*) for *spaCBA* and LGG_002372 (*spaF*), LGG_02371 (*spaE*), LGG_02370 (*spaD*), and LGG_02369 (*srtC2*) for *spaFED* [20]. Here, it is worth mentioning that based on an updated BlastP search of the NCBI database for homologs of these encoded gene products, *L. casei* and *L. paracasei* appear to be the only other types of lactobacilli also possessing the *spaCBA* and *spaFED* operons in their genomes (data not shown). Moreover, this is substantiated further by the results obtained from recent genomic-related studies involving these two *Lactobacillus* species [35], [42], [43].

Although each of the operon-encoded genes for both pilus types are expressible as soluble recombinant protein [24], so far the only published accounts of fully assembled cell-surface pili are those (called SpaCBA) constitutively expressed from the *spaCBA* operon in the *L. rhamnosus* GG strain [20], [45], [46] along with a few other strains as reported recently in Ref. [34]. Pili that are encoded by the *spaFED* operon (called SpaFED) have so far not yet been visualized on the outer surface of any *L. rhamnosus* strain [46], although a recombinant form of the *spaFED* operon-encoded pilus from *L. rhamnosus* GG can be assembled on the surface of lactococcal cells (our unpublished results). It is speculated that *L. rhamnosus* GG pilus gene expression is linked to an upstream “activating” insertion sequence (IS) element found in the *spaCBA* operon, but which is seemingly not present for the *spaFED*-related loci [47]. For the sortase-catalyzed assembly of the SpaCBA pilus in *L. rhamnosus* GG, repeating SpaA subunits form the polymerized backbone, and to which are added the basal SpaB subunit for cell wall anchoring and the tip SpaC subunit for adhesion [46]. To varying extents, these latter ancillary pilin subunits can also appear sporadically along the length of the pilus backbone structure [46]. Presumably taken as part of its possible functioning in the gut, the SpaCBA pilus can adhere to mucus [20], [24], collagen [25], and intestinal epithelial cells [23], stimulate the growth of biofilms [23], and modulate certain host immune-cell responses [23], [26]. From this, it is largely implicit that the adhesiveness of the SpaCBA pilus is then able to increase the transient longevity of *L. rhamnosus* GG cells in the GI tract, with the understood cellular effect of helping impart certain host health benefits.

Our pan-genome of the *L. rhamnosus* species indicates that the *spaCBA* operon is outside of the core genome, as it is not shared by all strains (Tables 2 and S1). Instead, the loci for the SpaCBA pilus are part of the dispensable genome, and in this context, they can be considered as rare genes in *L. rhamnosus*. In all, only four of the 13 genomes, each from strains with similar human gut-isolated origins (LMS2-1, E800, GG, and ATCC 53103), has the *spaCBA* operon present. An alignment of the primary structures for the three pilin subunits (SpaC, SpaB, and SpaA) and sortase protein (SrtC1) from these four genomes revealed only very slight differences at the amino acid level (Figures S1A–D). Of these, we were able to demonstrate that the *spaCBA* operon in the E800 strain is itself functionally active (as in the GG strain shown previously [20]) and able to direct surface expression of assembled pili (Figure S2). Taken as a functional advantage for the intestinal E800 strain, this would be helpful during niche adaptation by promoting enhanced cellular adhesion and gut colonization.

**Table 2:** Locus tags for functionally relevant surface-protein loci in the *L. rhamnosus* pan-genome.

Surface piliation (Locus tags)				
Strain	SpaCBA*		SpaFED**	
LC705	–		LC705_02369-02367	
ATCC 8530	–		LRHK_2379-2376	
GG	LGG_00444-00441		LGG_02372-02369	
ATCC 53103	LRHM_0428-0425		LRHM_2281-2078	
ATCC 21052	–		HMPREF0541_00153-00150	
HN001	–		LRH_09830-09845	
LMS2-1	HMPREF0539_0008-0005		HMPREF0539_2083-2086	
LRHMDP2	–		LRHMDP2_1747-1751	
LRHMDP3	–		LRHMDP3_2146-2142	
R0011	–		R0011_10665-10650	
E800	E800_215-218		E800_224-220	
PEL5	–		PEL5_2583-2580	
PEL6	–		PEL6_1769-1766	
**LPXTG-like surface protein (Locus tags)**				
**Strain**	**MBF** **		**MabA** **	
LC705	LC705_02328		LC705_01847	
ATCC 8530	LRHK_2337		LRHK_1841	
GG	LGG_02337		LGG_01865	
ATCC 53103	LRHM_2248		LRHM_1797	
ATCC 21052	HMPREF0541_03024		HMPREF0541_02074	
HN001	LRH_13741		R0011_04495	
LMS2-1	HMPREF0539_2127		HMPREF0539_1917	
LRHMDP2	LRHMDP2_1795		LRHMDP2_1316	
LRHMDP3	LRHMDP3_2005		LRHMDP3_697	
R0011	R0011_10445		LRH_01177	
E800	E800_1643		E800_2602	
PEL5	PEL5_2547		PEL5_2877^1^	
PEL6	PEL6_1733		PEL6_735	
**Surface-associated protein (Locus tags)**				
**Strain**	**Msp1** **	**Msp2** **		**Fbp** *
LC705	LC705_00310	LC705_00025		LC705_01467
ATCC 8530	LRHK_318	LRHK_28		LRHK_1451
GG	LGG_00324	LGG_00031		LGG_01450
ATCC 53103	LRHM_0311	LRHM_0032		LRHM_1393
ATCC 21052	HMPREF0541_00430	HMPREF0541_00213		HMPREF0541_00695
HN001	LRH_12224	LRH_09303		LRH_01637
LMS2-1	HMPREF0539_2610	HMPREF0539_0332		HMPREF0539_1504
LRHMDP2	LRHMDP2_732	LRHMDP2_2303		LRHMDP2_2187
LRHMDP3	LRHMDP3_2058	LRHMDP3_203		LRHMDP3_1981
R0011	R0011_07998	R0011_06697		R0011_02685
E800	E800_1025	E800_1407		E800_1845
PEL5	PEL5_2258	PEL5_2423		PEL5_1078
PEL6	PEL6_1525	PEL6_972		PEL6_2729

*Corresponding gene found in dispensable genome.

**Corresponding gene found in core genome.

1 ORF is non-concatenated sequence of two adjoining contigs.

Interestingly, however, although each of the four *spaCBA* operon-containing genomes are from strains with comparable gut origins, there were enough differences via inherent genomic plasticity (aside from those of GG and ATCC 53103) for them to be placed in unrelated positions in the reconstructed phylogenomic tree of *L. rhamnosus* (Figure 1). Of further interest, the genomic presence of *spaCBA*-related genes was not inclusive of all the intestinal isolates in the pan-genome (i.e., ATCC 21052, PEL5, and PEL6). Moreover, these pilus genes were also not associated with the genomes of strains recovered originally from the human oral cavity (LRHMDP2 and LRHMDP3) and respiratory tract (ATCC 8530). While it can be assumed that adapting to an epithelium-rich environment would seemingly favor the selection for mucus- and collagen-binding surface proteins such as the SpaCBA pilus, it was apparent that there was an inadequate evolutionary pressure for the prevalence of the *spaCBA* operon among the genomes of these other strains. Whether in fact this is reflected in either a loss or gain of pilus loci is not so obvious. However, just as it is that certain internal bodily micro-ecosystems are, by and large, open to the external environment, one can speculate that those strains without the *spaCBA* operon might themselves have arisen in the gut, mouth, or lungs more recently from other habitats. Similarly, for the three dairy-related strains (LC705, R0011, and HN001), despite being able to adapt to a milk-based environment that also includes mucins [48] and ECM proteins [49] shed from mammary epithelial cells, these isolates are lacking the *spaCBA* operon in their genomes and have no apparent reliance on a SpaCBA pilus-derived functionality.

Consistent in that this pan-genome there are only a few strains whose genomes have the required loci for SpaCBA piliation, it would seem reasonable to conclude the corresponding pilus genes might have been acquired as new genes by these particular intestinal *L. rhamnosus* isolates somewhat later during the niche adaptation process. Since the putative premise for LGT of the *spaCBA* operon in the *L. rhamnosus* GG genome has been proposed already previously [47], wherein the clustering of pilus genes is flanked on both sides by transposon-like IS elements, this then would seem a plausible event. As some indication of this, the coding region for the *spaC*, *spaB*, *spaA*, and *srtC1* genes in *L. rhamnosus* GG exhibit a slightly lower G+C content (45%, 45.3%, 44.6%, and 43.3%, respectively) than that of the whole genome (∼47%) [20], which suggests these loci might have been in the genome for a shorter period of (evolutionary) time, and so then acquired more recently. In this case, there could be a stronger likelihood for the possible lateral transfer of the pilus genes from the more densely populated gut microbiota than from among any indigenous piliated bacteria inhabiting the other fewer colonized niches. As an example, certain piliated gut-enterococci (*Enterococcus faecium* and *Enterococcus faecalis*) might have been the ancestral source for the *spaCBA*-related loci, as their pilin subunits show some primary structure similarities with those making up the SpaCBA pilus [20]. However, a possible conundrum arising here is whether *L. rhamnosus* GG and the other *spaCBA* operon-containing strains, all perceived as gut-transient bacteria, would have been able to survive long enough in the GI tract to evolve the LGT-driven genomic capacity for SpaCBA pili.

As another possible source of *spaCBA* genetic material, a recent pan-genome study of *L. casei* [42] (a species seen more so associated with dairy products and plants) has provided some convincing rationale that the *spaCBA* genes in the *L. rhamnosus* GG and LMS2-1 strains might have arisen originally from an earlier LGT event involving *L. casei*. In *L. casei*, which is considered a taxonomic close relative of *L. rhamnosus*, these pilus loci are so far common to all tested strains, but they apparently seem neither to have DNA flanking transposon-like elements [47] nor to have evolved recently in the genome [42]. Still, although cell-surface localization of SpaCBA pili is yet to be proven in *L. casei* (or in *L. paracasei* for that matter), the corresponding *spaCBA* operon can thus be considered a good candidate for the ancestral pilus genes in certain *L. rhamnosus* strains. Then again, as for the three intestinal strains lacking the *spaCBA*-related genes, it can be inferred that the continued presence of each in the gut is likely to have been too brief or recent evolution-wise for LGT to succeed. At any rate, those *L. rhamnosus* isolates with genomes encoding the SpaCBA pilus have made the necessary genomic adjustments for the capacity to persist less transiently in the gut, and, in so doing, have ensured these strains are more competitively advantaged and then able to survive and colonize longer in this particular environmental niche. In the context of so-perceived beneficial activity, the SpaCBA-piliated strains would then seem to be better positioned for modulating host cell immune responses in the gut than any of those strains lacking such piliation.

Regarding the *spaFED* operon, the *L. rhamnosus* pan-genome shows that these pilus genes are common to all the strains and isolates (Tables 2 and S1) and then to be included in the core genome. Based on our own results involving two gut (GG and E800) and two dairy-related (LC705 and R0011) strains (data not shown), and as well to the best of our knowledge, a *L. rhamnosus* isolate has yet to be shown to exhibit cell surface-localized SpaFED pili, and thus to date they remain elusive and hypothetical surface structures in this species. Here, as possible reasons for being dormant, it can be surmised that a specific inducible promoter perhaps controls the triggered transcription of the *spaFED* genes, or otherwise the corresponding regulatory sequence region for constitutive expression has somehow become deleted or corrupted when this operon was acquired. On the other hand, it can be mentioned that with a set of pilus loci that is presumably not readily transcribed, any habitat wherein these genes should then become inducibly expressed likely must harbor an exclusive signaling stimulus, but also then still be somewhat widespread.

Speculatively, as an unexpressed *spaFED* operon seemingly poses no liability to the *L. rhamnosus* genomes (strains), it would still not be offering any sort of advantage or fitness benefit to cells. So with that, it is unclear why these loci are a constant feature and remain present in the genomes of the *L. rhamnosus* strains, particularly those containing the *spaCBA* operon, and instead not have succumbed to decay and loss by genomic evolutionary forces. In fact, *spaFED* genes are present in all the sequenced genomes of *L. casei* and *L. paracasei* [42], [43], but here as well in these species they remain to be confirmed as cell surface-expressed/assembled structures. By comparison, those genes of the *L. rhamnosus* GG *spaFED* operon (*spaF*, *spaE*, *spaD*, and *srtC2*) appear to have a somewhat higher G+C content (49.3%, 48.2%, 48.9%, and 53.7%, respectively) than those of the *spaCBA* operon and the rest of the genome (see above). Here, this might mean the *spaFED* genes are more stable and better at withstanding certain environmental stresses at the DNA level. Relative to the *spaCBA* operon, this is perhaps suggestive of a much earlier genomic acquisition of the *spaFED* genes, which most likely had transpired by LGT, and then possibly involved *spaFED*-containing *L. casei* or *L. paracasei*. If further speculation is taken, and this cluster of genes is in fact lying dormant in the genome, there can also be the prospect that the *spaFED* operon is an evolutionary remnant from a distant ancestral species (e.g., *E. faecium* or *E. faecalis*), but which nonetheless is maintained in *L. rhamnosus* (and as well *L. casei* and *L. paracasei*) for an unknown reason or intended purpose.

#### Mucus-binding factor (MBF) protein

In the *L. rhamnosus* GG genome, only a single ORF encodes a protein product that shares any amino acid identity with known mucus-binding domains [20]. Here, the primary structural elements in the gene (LGG_02337 or *mbf*) are an N-terminal secretion signal, four Pfam-MucBP (mucin-binding protein) domain repeats, and a C-terminal LPXTG-like cell wall-anchoring domain [21]. As a mature ∼38-kDa surface protein, recently renamed the mucus-binding factor (MBF) to correct an earlier misannotation as an internalin, it represents another of the cell wall-anchored adhesins that has been characterized as functionally mucoadhesive in *L. rhamnosus* GG cells, but then less so than the mucus-binding SpaCBA pilus [21]. Moreover, a protein homolog of MBF in *L. rhamnosus* LC705 has also been reported [20], although in this non-SpaCBA-piliated and less mucus-adherent strain [50], it appears to be the key participatory mucus-interacting surface adhesin [21]. An updated BlastP search against the NCBI database has revealed proteins with the greatest level of sequence similarity to MBF are to be found in other *L. rhamnosus* strains and as well at lesser levels in taxonomic related *L. casei* and *L. paracasei* (data not shown). However, also apparent are distant levels of similarity to proteins in *Listeria*, *Enterococcus*, *Pediococcus*, and *Lactococcus* species (data not shown).

Our pan-genome comparison of the *L. rhamnosus* genomes shows that there is no variation in the occurrence the *mbf* locus among the different strains, and so this gene is included in the core genome (Tables 2 and S1). The common presence of a genotype encoding a mucoadherent phenotypic trait in the 13 genomes is fully consistent with the isolated-origin of the various *L. rhamnosus* strains. Here, the strains isolated from inner body regions layered with a mucosal epithelium (i.e., GI and respiratory tracts and oral cavity) would find having a functional mucus-specific surface adhesin both advantageous and necessary for any level of colonization. Likewise, those *L. rhamnosus* strains recovered from a milk-based milieu would also similarly benefit from the specificity of the MBF surface protein. In this respect, it is conceivable that the presence of milk mucins [48] would have provided the evolutionary pressure to maintain the *mbf* gene in the genomes of these dairy-related strains and, in particular, any of those *L. rhamnosus* strains whose genomes do not carry genes for the mucoadhesive SpaCBA pilus. Based on the mucus-binding data reported previously for the milk-isolated *L. rhamnosus* LC705 strain [50], it would seem applicable that all those other strains missing the genomic capacity for SpaCBA piliation are then more likely to be poorer binders of mucus. In addition, as an alignment of the MBF primary structures shows only a few shared amino acid substitutions (>96% overall identity), one can surmise that given the negligible strain-related variation at the amino acid level (Figure S1E), the mucus-binding properties for the corresponding MBF proteins are then similar. Consequently, for any of those non-piliated strains it is likely that an *mbf*-expressed and surface-localized protein might then help reinforce a presumed gut-transient colonization behavior. Moreover, as an active MBF will thus no doubt sustain some level of mucoadhesiveness in *L. rhamnosus* cells, this then can contribute to the respective habitation of the various strains included in our pan-genome construction.

#### Modulator of adhesion and biofilm (MabA) protein

Another protein identified in *L. rhamnosus* GG as being adhesive is known as the modulator of adhesion and biofilm (MabA) protein [22], and in the genome it is encoded by the LGG_01865 (or *mabA*) ORF [20]. Because the primary structure includes a LPXTG-like motif at the C-terminus, this rather large ∼250-kDa protein is more likely to be cell wall-anchored than secreted and released. Additionally, its primary structural organization consists of 26 domain repeats of unknown function (DUF1542), each of which is about 75 amino acids in length. The DUF1542 domain is itself also present in various other cell-surface proteins, with some of these being implicated in cellular adhesion and antibiotic resistance. As its purported function in *L. rhamnosus* GG, the MabA protein has been associated with the capacity to bind intestinal epithelial Caca-2 cells and stimulate biofilm growth [22]. BlastP search results indicate that potential homologs of the *L. rhamnosus* GG MabA protein with the best sequence identities are mainly found in other *L. rhamnosus* strains (>94%) and the bovine milk-isolated *L. zeae* KCTC 3804 strain (36 to 61%), but interestingly as well, in some pathogenic *Streptococcus pyogenes* strains (35 to 38%) (data not shown). Moreover, whereas related homologs in streptococci also have an extra N-terminal domain specifying an ECM-binding capacity [51], this particular feature is not associated with *L. rhamnosus* GG MabA [22]. However, beyond this, minimal is known about the functional role played by any of the “MabA-like” proteins in different bacterial species.

As shown from our results, strain variation within the *L. rhamnosus* pan-genome for the *mabA* locus is absent (Tables 2 and S1). Based on this, this adhesion-related gene is included in the makeup of the core genome. Given that little is actually understood about the *mabA*-expressed product and its functional phenotype, any correlation with the isolated-origins of the different strains is then rather more problematic. Intriguingly, even though the MabA protein is clearly seen as a common feature of the *L. rhamnosus* species, some notable variability at the amino acid level for this presumed adhesin exists among the different strains. For instance, based on primary structural alignments, major differences at both the N- and C-terminal regions, along with numerous single-site substitutions, are found in the various MabA homologs (Figure S1F). (For the PEL5 genome, it should be noted that because a short stretch of unsequencable DNA is in the gap between the two adjoining contigs that contain the corresponding ORF, i.e., PEL5_2877, “X” then represented some of the internal primary structure for the predicted protein.) Functionally, there is no obvious connection between this primary structure variability and the habitats these strains had been isolated originally. Nonetheless, as the *mabA* gene is part of the *L. rhamnosus* core genome, the encoded MabA protein, via its purported properties, might then be understood as some benefit to this species when adapting to certain niches, for instance, like the gut. Moreover, although the streptococcal ECM-binding counterpart of MabA can be viewed as a possible virulence factor, one can speculate that its associated gene might have been the predecessor to the *mabA* gene in the *L. rhamnosus* species, possibly having arisen by LGT, but with the corresponding MabA protein then modified to instead function as a more harmless surface-related niche-adaptation factor.

#### Msp1/Msp2 (p75/p40) proteins

Included among the genome-predicted cell wall-associated proteins in *L. rhamnosus* GG are the two “major-secreted-proteins”, Msp1 (or p75) and Msp2 (or p40) [20]. Each of these proteins (about 47 and 42 kDa in size for Msp1 and Msp2, respectively) is loosely attached to the cell surface, but as well is released from cells [27]. Genomically, the LGG_00324 and LGG_00031 genes respectively encode the Msp1 and Msp2 proteins [20]. Here, both genes are predicted as modular proteins, with each having an N-terminal domain of no known function, but then displaying homology to the peptidase NLPC/P60 [PF00877] (for Msp1) and CHAP [PF05257] (for Msp2) domains at the C-terminus end [27], [52]. Related to the latter domain predictions, peptidoglycan hydrolase activities have been demonstrated for each protein, with Msp1 (confirmed as glycosylated in *L. rhamnosus* GG [53]) exhibiting D-glutamyl-L-lysyl endopeptidase activity [52] and Msp2, as a homolog version in *L. casei*, being able to degrade cell wall muropeptides [54]. However, as a rather noteworthy earlier finding [55], both Msp1 (p75) and Msp2 (p40) were shown to behave as so-called “moonlighting” proteins and, among reported immune functions, are able to help preclude cytokine-induced intestinal epithelial cell damage and death through EGF (epidermal growth factor) receptor-dependent activation of anti-apoptotic PI3K/Akt signaling [27]–[30].

Based on our pan-genome of *L. rhamnosus*, there was no genetic variation for the Msp1- and Msp2-related loci among the 13 genomes, thus indicating them to be common core genes in the different strains (Tables 2 and S1). This is somewhat understandable in view of how well conserved each of the two proteins is at the amino acid level. Here, a primary structure alignment of Msp2 shows that among the various strains this protein exhibits >99.5% overall identity, with only one single-site substitution being present near the N- and C-terminus ends (Figure S1H). Similarly, when the predicted amino acid sequences of Msp1 are aligned, there is >99% overall identity (Figure S1G). However, in addition to a few mid-to-end C-terminal single-site amino acid substitutions, genomes from a few of the strains encode predicted Msp1 proteins that encompass a 5- or 15-residue deletion at a position just midway through the primary structure (Figure S1G). As such, even though Msp1 and Msp2 both behave as “non-adhesive” cell-surface phenotypic traits, their core-genome genotypes, together with the strongly conserved primary structures of these two proteins, seems to underscore the functional and structural importance as active cell wall-related hydrolases during normal cell division and separation. This in itself would then make the Msp1 and Msp2 proteins indispensable for the niche colonization and survival of the *L. rhamnosus* species as a whole, but as a result not give any type of adaptive preference to one or another of the different habitats. In such a context, the moonlighting activities of these two proteins in *L. rhamnosus* (see above) will then offer a universal “health” benefit to any naturally or otherwise colonized human and animal hosts.

#### Fibronectin-binding protein (Fbp)

Amongst the surface-associated ECM glycoproteins of epithelium-like cells there is fibronectin, which in itself serves as one of the prime binding targets for many pathogens, but as well as an attachment site for numerous commensal bacteria, like the lactobacilli [56]. Here, various fibronectin-binding proteins (Fbp) encoded in the genomes of these bacteria are known for helping mediate this form of adherence to the epithelial layers of the gut, oral and vaginal cavities, and breathing airways and lungs [56]. Significantly, present in the *L. rhamnosus* GG genome is the gene (LGG_01450 or *fbp*) for a predicted ∼64-kDa protein [20] whose homolog (so-called FbpA) in the taxonomically close *L. casei* species has had previously been characterized in its recombinant form as functionally adhesive to fibronectin [57]. However, unlike other fibronectin-binding proteins in pathogens, the *L. casei* FbpA protein has neither a recognizable N-terminal secretion signal nor an obvious C-terminal cell wall-anchoring motif [57], two features that are as well absent from the primary structure of the LGG_01450-encoded protein in *L. rhamnosus* GG [20]. Consequently, this type of fibronectin adhesin is viewed as somewhat uncharacteristic, and for *L. casei* it was shown to be only weakly associated with the cell surface [57].

Analysis of the *L. rhamnosus* pan-genome shows that the gene homolog of the *L. rhamnosus* GG fibronectin-binding protein (LGG_01450) is present in all 12 other genomes examined (Tables 2 and S1). Accordingly, the so-called *fbp* gene is part of the core genome of this species, as it is common to the different strains and most likely then functionally relevant in the context of their isolated origins. However, as none of the *fbp*-encoded proteins was detected by the SignalP program, and so not among the predicted proteins in our pan-secretomic survey, it would appear that all are lacking a classical signal sequence at the N-terminus of their primary structures. Nonetheless, owing to the endogenous presence of fibronectin in epithelial cells, evolutionary selection for the *fbp* locus in the core genome is rather expected for those strains isolated from the human GI and respiratory tracts as well as from intestinal biopsies and dental infections. Similarly, this would seem the case for also the dairy-related strains given that fibronectin is produced by mammary epithelial cells [49], and so this then makes it potentially available as a binding substrate in milk and thereafter any milk-derived products. Because a primary structure alignment of Fbp from these various strains demonstrates >98% overall identity (Figure S1I), as a surface-associated adhesin, it can likely be considered structurally and functionally conserved in *L. rhamnosus*. Here, in the context of actual *fbp* gene expression, there is a common advantage to having the genotype for a fibronectin-binding phenotype in each of these different *L. rhamnosus* strains, and as such would be one of another of the outer surface-related features that could help facilitate a particular lifestyle habitation.

## Concluding Comments

It is with little doubt that the manner in which a bacterial cell makes contact and physically interacts with the host environment is largely dependent on the diverse composition and character of its outer-surface proteins. As a means to explore the genotype-phenotype variability between *L. rhamnosus* strains at the cell-surface level, and as well how this might relate to any preference in ecological adaptability to mixed habitats, we took a purely pan-genomic viewpoint and focused our efforts on certain functionally relevant surface-associated proteins. Here, the extent to which this pan-genome is comprised of rare or common genes specifying functional cell wall-localized proteins has let us gauge which of the outer surface features might represent an advantage for adapting to certain host niches. In this regard, and amongst the various different types of outer surface proteins we had set forth to scrutinize genomically, one of our most noteworthy findings dealt with the cluster of genes needed for assembling the SpaCBA-called pilus surface appendage. Based on our pan-genome survey of *L. rhamnosus*, it was clear to us that the corresponding *spaCBA* operon can be considered a genomic rarity in this species of LAB as evidenced by its inclusion in the dispensable genome, but with that, whose presence can lead to the advantageous functional mucus-binding usage in certain strains. Presumably, this phenotypic trait would be for promoting a protracted transient colonization of the gut epithelium, and thus the *spaCBA* operon can then be viewed as advancing the niche-specific fitness of any so-piliated strains (e.g., *L. rhamnosus* GG and E800). However, it was also obvious from the pan-genome that in spite of the SpaCBA pilus being perceived as a useful niche-adaptation factor for gut-dwelling *L. rhamnosus* strains, not all genomes of strains isolated from the intestinal milieu have the *spaCBA* operon present. Moreover, this is also the situation for those strains originally isolated from the mucosal-lined oral cavity and respiratory airways, and as well, those being recovered from a milk-based environment. Here, with the omnipresence of the *spaCBA* operon lacking in these other strains, we surmise this reflects certain environmental variances in the individual habitats that lessen the apparent need for such mucoadhesive functionality and in effect negate the acquirement of any SpaCBA pilus genes through LGT-related activity.

Of the remaining outer-surface proteins that we focused on in this study, their confirmed and/or projected roles are varied and include mucus and fibronectin adhesion, biofilm formation, peptidoglycan hydrolysis, and host immune responsiveness. Our pan-genome data indicates each of the corresponding encoded ORFs is part of the *L. rhamnosus* core genome. Accordingly, these are to be considered common or, in effect, housekeeping-type genes, and with that, one might reason that the physiological functioning of each different protein (i.e., MBF, MabA, Msp1, Msp2, Fbp, and more speculatively SpaFED) is indispensable to the overall outward cellular action of the *L. rhamnosus* strains and therefore essential during specific surface-related interactions with host and environment. In fact, most of the loci for these various surface-associated proteins had also been found in the core genomes of *L. casei* [42] and *L. paracasei* [43], which then further reinforces the functional universality of such proteins in three close-related lactobacilli species. With that being said, for the *L. rhamnosus* species in particular, it would seem certain there is a commonality of selective pressures amongst the niche habitats that then generates the needed evolutionary forces for genetically sustaining each of these “core-type” surface-associated proteins in strains of various origins.

## Materials and Methods

### Ethics statement

The research proposal to retrieve and use human intestinal biopsy specimens for the recovery of *L. rhamnosus* isolates (PEL5 and PEL6) had been approved by the chief of the surgical clinic as according to the ethical rules and guidelines valid for the sampling year (1997) at the Jyväskylä District Central Hospital. Pertaining to this, the two patient-donors involved had signed their informed consent beforehand.

### *L. rhamnosus* strains

*L. rhamnosus* PEL5 and PEL6 strains were clinical isolates of biopsy specimens (Jyväskylä Central Hospital and Institute of Clinical Medicine, Jyväskylä, Finland) removed from the colon and caecum, respectively, of two otherwise healthy individuals having some intestinal problems. For pure culture isolation of *L. rhamnosus*, the two intestinal biopsy specimens were provided to the microbiology research laboratory at MTT Agrifood Research Finland (Jokioinen, Finland) as anonymous samples, with the use of standard isolation protocols and methods under anaerobic conditions then following. Pure cultures were verified initially by biochemical characterizations and API 50 CHL tests, and subsequently by 16S rRNA sequence analysis and ribotyping. The *L. rhamnosus* E800 (VTT E-97800) strain was isolated originally from adult feces [58] and acquired for this study from the VTT culture collection (http://culturecollection.vtt.fi/; VTT Technical Research Center of Finland, Espoo, Finland).

### Genome sequencing and annotation

Genomes from *L. rhamnosus* strains PEL5, PEL6, and E800 were sequenced at the Institute of Biotechnology (University of Helsinki, Finland) using next-generation sequencing platforms. For this, cells of each strain were grown and harvested by procedures described previously [20], and from which the corresponding genomic DNA was then recovered using commercial DNA extraction kits. Genomic DNAs were then fragmented in a microTube (3 µg per 100 µl) using a Covaris S2 acoustic shearing device (Covaris Inc., USA). For a 50-µl volume sample, fragment length separation to a DNA size of 1.2 kb using magnetic carboxyl beads was carried out similarly as described previously [59]. After the size-selection step, DNA fragments were subjected to end polishing and 454 Y-adapter ligation according to the protocols provided by the manufacturer (Roche/454 Life Sciences, USA). After the genomic libraries were amplified by emulsion PCR, they were run on the Roche Genome Sequencer FLX+ system, with the sequence contigs then being assembled *de novo* using the GS Assembler (Roche/454 Life Sciences, USA). These three newly-sequenced genomes were deposited in GenBank under the accession numbers JDFQ00000000 (PEL5), JDFR00000000 (PEL6), and JDRW00000000 (E800). Ten additional good-quality sequenced/annotated *L. rhamnosus* genomes were obtained from the NCBI RefSeq database (as of June 2013). For the annotation process, assembled DNA sequences of the new draft genomes from the PEL5, PEL6, and E800 strains (but as well as from the LRHMDP2 and LRHMDP3 strains) were run through an automatic annotation pipeline via RAST (Rapid Annotation using Subsystem Technology) [60], followed by some extra manual curation.

### Orthologous gene prediction and genome sequence comparison

For identifying the orthologous genes among the 13 *L. rhamnosus* genome sequences, a BlastP algorithm [61] with a default score matrix (BLOSUM62) and an *E*-value cut-off of 1 × 10^−04^ was used in an all-versus-all gene-to-gene comparison involving every genome. Scoring for each BLAST hit was normalized against the maximum possible score, which is defined as the self-hit score for the query gene. This gives BLAST score ratio values (SRV) that range from 0 to 100, and so are then more reflective of the “hit quality” than are the raw BLAST bit-score values [62]. Accordingly, two genes are then identified as orthologs if they mutually have a pair-wise reciprocal best BLAST hit (RBBH), and where the SRV exceeds 35 for both single hits.

Using the above criteria, the orthologous relationships were then defined for the *L. rhamnosus* pan-genome as well as for the core and dispensable genomes. For estimating the pan-genome, an additive approach was used, in which through comparisons of the genomes from the various strains, different sets of non-orthologous genes are sequentially added to the set of genes from the strain (*L. rhamnosus* GG) arbitrarily chosen as the basis for building up the pan-genome. The core genome was instead estimated by a series of reductive comparisons between the strain-genomes and includes a set of genes that are orthologs in all 13 *L. rhamnosus* genomes. The dispensable genome includes all the non-orthologous genes in the pan-genome, and in effect are those genes shared by at least two but not all *L. rhamnosus* genomes. As subsets of the dispensable genes, there are the unique (or strain-specific) genes, defined as those genes that are only present in a single strain-genome, and the ORFan-like genes, defined as those genes in the different *L. rhamnosus* genomes that lack a homologous match in the *Lactobacillus* spp. genome database. All comparative analyses performed for obtaining orthology-related data had utilized the EDGAR software platform [63]. Pan-genome development of *L. rhamnosus* was calculated with R statistical programming language and using Heap’s Law, wherein the parameter values (κ, γ, and α) were approximated from a plotted curve representing the nonlinear least squares fit of the genome data [36]–[37].

The *L. rhamnosus* pan-secretome of classically secreted or exported proteins was constructed based on the algorithmic predictions of the SignalP 4.1 program (http://www.cbs.dtu.dk/services/SignalP/) using default settings (cut-off score of 0.45 or greater) for Gram-positive bacteria [64]. Here, the pan-secretome is derived from loci that encode for those putative proteins having a classical N-terminal signal peptide sequence (as predicted by SignalP), with these being identified and then pooled collectively after successive additive comparisons of all 13 *L. rhamnosus* genomes. The corresponding core and dispensable secretome proteins were the predicted products of orthologous and non-orthologous genes, respectively.

### Phylogenomic tree reconstruction

To establish the evolutionary relationships among the various *L. rhamnosus* strains (genomes) we built a phylogenomic tree using methodologies described previously [65]. Briefly, phylogenetic tree reconstruction was based on the approach used by Ref. [66], in which predicted protein homologies were used to identify possible genes in each of the different genomes. Here, multiple genome alignments of mutually conserved orthologous genes from the core genome were produced with MUSCLE [67]. After the blocks of multiple alignments were concatenated, any gappy or misaligned regions were then removed with GBLOCKS [68]. Phylogenies based on these alignment data were generated using the neighbor-joining algorithm in the PHYLIP package [69].

## Supporting Information

Figure S1(A–I) **Primary structure comparison of surface-protein homologs of** ***L. rhamnosus*** **.** Predicted homolog sequences of the SpaC (**A**), SpaB, (**B**), SpaA (**C**), SrtC1 (**D**), MBF (**E**), MabA (**F**), Msp1 (**G**), Msp2 (**H**), and Fbp (**I**) proteins were extracted from the following *L. rhamnosus* genomes: LC705, ATCC 8530, GG, ATCC 53103, ATCC 21052, HN001, LMS2-1, LRHMDP2, LRHMDP3, R0011, E800, PEL5, and PEL6. Corresponding locus tags for each respective gene/protein are provided in Table 2. Individual multiple alignments of the amino acid sequences for each type of protein were done using the MultAlin program [70] (http://multalin.toulouse.inra.fr/multalin/multalin.html). Residues matching exactly the consensus sequence (defined as the amino acids found in 100% of the sequences) are marked in red, whereas those deviating from the consensus sequence are marked in black. Positions in the consensus sequence that denote conservative amino acid replacements are indicated by symbols (!, #, or $).
(PDF)

Figure S2 **Immunoblotting confirmation of** ***spaCBA*** **-encoded pilus production in** ***L. rhamnosus*** **E800.** Whole cells of overnight-grown *L. rhamnosus* E800 were run on SDS-polyacrylamide gels and SpaA-containing SpaCBA pili were detected by immunoblotting (lane 2) with rabbit polyclonal antiserum specific for recombinant *L. rhamnosus* GG SpaA pilin protein [24] using procedures described previously [46]. SpaCBA-piliated *L. rhamnosus* GG cells were treated similarly and used as the positive control (lane 1). Different-lengthened pili are represented by a dense laddered region of high-molecular-weight (HMW) protein bands, and are so indicated on the top right of the immunoblot. An asterisk on the bottom right identifies the approximate location of monomeric SpaA pilin protein (∼30 kDa). Sizes and positions of the molecular weight markers are shown on the left.
(TIF)

Table S1 **Predicted core and dispensable genes of the** ***L. rhamnosus*** **pan-genome.** Listed are the predicted loci from the 13 genomes that make up the core (2095) and dispensable (2798) parts of the *L. rhamnosus* pan-genome (4893). Locus tags of genes (with specified annotations) that belong to the core (yellow highlight) and dispensable genomes are listed. Locus tags for the 75 core genes (per each *L. rhamnosus* genome) that are not found in any other *Lactobacillus* species are indentified by red font. Locus tags for the unique (blue font) and ORFan-like (grey highlight) genes in the dispensable genome are indicated.
(XLSX)

Table S2 **Predicted loci encoding the “classical” pan-secretome of** ***L. rhamnosus*** **.** Provided is a list of predicted loci from 13 genomes that encode the core (103) and dispensable (127) proteins of the *L. rhamnosus* pan-secretome (230). Locus tags of genes (with specified annotations) belonging to the core (blue highlight) and dispensable (orange highlight) secretome are listed.
(XLSX)
