# Supplementary material for: A Comparative Pan-Genome Perspective of Niche-Adaptable Cell-Surface Protein Phenotypes in Lactobacillus rhamnosus
Source: PLoS One. 2014 Jul 17;9(7):e102762. doi: 10.1371/journal.pone.0102762 (PMC4102537; doi:10.1371/journal.pone.0102762)
Supplement: Figure S1(A–I) — Primary structure comparison of surface-protein homologs of L. rhamnosus . Predicted homolog sequences of the SpaC (A), SpaB, (B), SpaA (C), SrtC1 (D), MBF (E), MabA (F), Msp1 (G), Msp2 (H), and Fbp (I) proteins were extracted from the following L. rhamnosus genomes: LC705, ATCC 8530, GG, ATCC 53103, ATCC 21052, HN001, LMS2-1, LRHMDP2, LRHMDP3, R0011, E800, PEL5, and PEL6. Corresponding locus tags for each respective gene/protein are provided in Table 2. Individual multiple alignments of the amino acid sequences for each type of protein were done using the MultAlin program [70] (http://multalin.toulouse.inra.fr/multalin/multalin.html). Residues matching exactly the consensus sequence (defined as the amino acids found in 100% of the sequences) are marked in red, whereas those deviating from the consensus sequence are marked in black. Positions in the consensus sequence that denote conservative amino acid replacements are indicated by symbols (!, #, or $). (PDF) [file pone.0102762.s001.pdf]

A

```
           1       10        20        30        40        50        60        70        80        90        100       110       120      130
GG            MTAKVARTGHLFAVLLILMSMLTGLVTSGSSVVTATDNIRPTYQTDANGTYPTNSWQVTGQQNVINQRGGDQVSGWDNNTIWNGDATDTTNSYLKFGDPNNPDYQIRKYAKETNTPGLYDVYLNVKG
ATCC53103     MTAKVARTGHLFAVLLILMSMLTGLVTSGSSVVTATDNIRPTYQTDANGTYPTNSWQVTGQQNVINQRGGDQVSGWDNNTIWNGDATDTTNSYLKFGDPNNPDYQIRKYAKETNTPGLYDVYLNVKG
LMS2-1     VTKMTAKVARTGHLFAVLLILMSMLTGLVTSGSSVVTAAANIRPTYQTDANGTYPTNSWQVTGQQNVINQRGGDQVSGWDNNTTWDGDATNTTNSYLKFGDPNKPDYQIRKYAKETNTPGLYDVYLNVKG
E800       VTKMTAKVARTGHLFAVLLILMSMLTGLVTSGSSVVTAAANIRPTYQTDANGTYPTNSWQVTGQQNVINQRGGDQVSGWDNNTTWDGDATNTTNSYLKFGDPNNPDYQIRKYAKETNTPGLYDVYLNVKG
Consensus  ...MTAKVARTGHLFAVLLILMSMLTGLVTSGSSVVTA..NIRPTYQTDANGTYPTNSWQVTGQQNVINQRGGDQVSGWDNNT.W#GDAT#TTNSYLKFGDPN.PDYQIRKYAKETNTPGLYDVYLNVKG

           131     140       150       160       170       180       190       200       210       220       230       240       250      260
GG         NKQQNVKPVDIVLVVDMSGSMESNRWGTNRAGAVRTGVKNFLTSIQNAGLGNYVNVGLIGFSSPGYIGGKSGYISVKLGKAGNASQQQAINGALSPRFQGGTYTQIGLRQGSAMLNADTSGNKKMMILLT
ATCC53103  NKQQNVKPVDIVLVVDMSGSMESNRWGTNRAGAVRTGVKNFLTSIQNAGLGNYVNVGLIGFSSPGYIGGKSGYISVKLGKAGNASQQQAINGALSPRFQGGTYTQIGLRQGSAMLNADTSGNKKMMILLT
LMS2-1     NTQQNVKPVDIVLVVDMSGSMESNSSGTNRAGAVRTGVKNFLTSIQNAGLGNYVNVGLIGFSSPGYIGGKSGYISVKLGKAGNASQQQAINGALSPRFQGGTYTQIGLRQGSAMLNADASGNKKMMILLT
E800       NTQQNVKPVDIVLVVDMSGSMESNSSGTNRAGAVRTGVKNFLTSIQNAGLGNYVNVGLIGFSSPGYIGGKSGYISVKLGKAGNASQQQAINGALSPRFQGGTYTQIGLRQGSAMLNADASGNKKMMILLT
Consensus  N.QQNVKPVDIVLVVDMSGSMESN..GTNRAGAVRTGVKNFLTSIQNAGLGNYVNVGLIGFSSPGYIGGKSGYISVKLGKAGNASQQQAINGALSPRFQGGTYTQIGLRQGSAMLNAD.SGNKKMMILLT

           261     270       280       290       300       310       320       330       340       350       360       370       380      390
GG         DGVPTFSNEVINSEWINGTLYGTNFGSSRDEPGNTARLRWPYTDSSGHYIYDTWPATLGEAKIAKDSGNEVHALGIQLADDDHYMTKEKIRQNMQLITNSPDLYEDADSADAVEAYLNNQAKDIIKNFNT
ATCC53103  DGVPTFSNEVINSEWINGTLYGTNFGSSRDEPGNTARLRWPYTDSSGHYIYDTWPATLGEAKIAKDSGNEVHALGIQLADDDHYMTKEKIRQNMQLITNSPDLYEDADSADAVEAYLNNQAKDIIKNFNT
LMS2-1     DGVPTFSNEVINSEWINGTLYGTNFGSSRDEPGNTAQLGWPYIDSSGNRIYDTWPATLGEAKKAKDSGNEVHALGIQLADDRKYMTKEKIRQNMQLITNSPDLYEDADSADAVEAYLNNQAKDIIKNFNT
E800       DGVPTFSNEVINSEWINGTLYGTNFGSSRDEPGNTAQLGWPYIDSSGNRIYDTWPATLGEAKKAKDSGNEVHALGIQLADDRKYMTKEKIRQNMQLITNSPDLYEDADSADAVEAYLNNQAKDIIKNFNT
Consensus  DGVPTFSNEVINSEWINGTLYGTNFGSSRDEPGNTA.L.WPY.DSSG..IYDTWPATLGEAK.AKDSGNEVHALGIQLADD..YMTKEKIRQNMQLITNSPDLYEDADSADAVEAYLNNQAKDIIKNFNT

           391     400       410       420       430       440       450       460       470       480       490       500       510      520
GG         VTDGTITDPIGTQFQYANNQATVTSVGKQTVPASELPSAAIQDGQLTVNHMNLGQDQEVQIHYQVRIKTEDAGFKPDFWYQMNGETLLTPKAGAAAVDFGIPSGRAPATTVYVVQKQWRQLSNQSLPDTLN
ATCC53103  VTDGTITDPIGTQFQYANNQATVTSVGKQTVPASELPSAAIQDGQLTVNHMNLGQDQEVQIHYQVRIKTEDAGFKPDFWYQMNGETLLTPKAGAAAVDFGIPSGRAPATTVYVVQKQWRQLSNQSLPDTLN
LMS2-1     VTDGTITDPIGTQFQYANNQTTVTSVGKQAVPASELPSAAIQDGQLTVNHMNLGQDQEVQIHYQVRIKTEDAGFKPDFWYQMNGETLLTPKAGAAAVDFGIPSGRAPATTVYVVQKQWRQLSNQSLPDTLN
E800       VTDGTITDPIGTQFQYANNQTTVTSVGKQAVPASELPSAAIQDGQLTVNHMNLGQDQEVQIHYQVRIKTEDAGFKPDFWYQMNGETLLTPKAGAAAVDFGIPSGRAPATTVYVVQKQWRQLSNQSLPDTLN
Consensus  VTDGTITDPIGTQFQYANNQ.TVTSVGKQ.VPASELPSAAIQDGQLTVNHMNLGQDQEVQIHYQVRIKTEDAGFKPDFWYQMNGETLLTPKAGAAAVDFGIPSGRAPATTVYVVQKQWRQLSNQSLPDTLN

           521     530       540       550       560       570       580       590       600       610       620       630       640      650
GG         VTVQRKVADGSLDPNWQQTLVLKKADNWKASFTAPAYNNQGQSFSYVVKSEDASGIDLSSFISSQNMDQQTATLTLTNQQYGFQFQKKTTDGTDLSADQLKAMQFNLTQYSDNSFQQASKTNAITSTDLQ
ATCC53103  VTVQRKVADGSLDPNWQQTLVLKKADNWKASFTAPAYNNQGQSFSYVVKSEDASGIDLSSFISSQNMDQQTATLTLTNQQYGFQFQKKTTDGTDLSADQLKAMQFNLTQYSDNSFQQASKTNAITSTDLQ
LMS2-1     VTVQRKVADGSLDPNWQQTLVLKKADNWKASFTAPAYNNQGQSFSYVVKSEDASGIDLSSFISSQNMDQQTATLTLTNQQYGFQFQKKTTDGTDLSTDQLKAMQFNLTQYSDNSFQQASKTNAITSTDLQ
E800       VTVQRKVADGSLDPNWQQTLVLKKADNWKASFTAPAYNNQGQSFSYVVKSEDASGIDLSSFISSQNMDQQTATLTLTNQQYGFQFQKKTTDGTDLSADQLKAMQFNLTQYSDNSFQQASKTNAITSTDLQ
Consensus  VTVQRKVADGSLDPNWQQTLVLKKADNWKASFTAPAYNNQGQSFSYVVKSEDASGIDLSSFISSQNMDQQTATLTLTNQQYGFQFQKKTTDGTDLS.DQLKAMQFNLTQYSDNSFQQASKTNAITSTDLQ

           651     660       670       680       690       700       710       720       730       740       750       760       770      780
GG         ALAPGYYGIQEAAAPTGYQLDGTTYLFQLTSDGQWQYHGTKDNVTSGSVINGQQTLNPVGDKSDDFTVTGDHQQILTLTKYDEPKPSMTLRVIKQDNQSQYLAGAAFTLQPSAGEAETITSSATSEGQAF
ATCC53103  ALAPGYYGIQEAAAPTGYQLDGTTYLFQLTSDGQWQYHGTKDNVTSGSVINGQQTLNPVGDKSDDFTVTGDHQQILTLTKYDEPKPSMTLRVIKQDNQSQYLAGAAFTLQPSAGEAETITSSATSEGQAF
LMS2-1     ALAPGYYSIQEAAAPTGYQLDGTTYLFQLTSDGQWQYHGTKDNVTSGSVINGQQTLNPVGDKSDDFTVTGDHQQILTLTKYDEPKPSMTLRVIKQDNQTQYLAGAAFTLQPSAGEAETITSSATSEGQAF
E800       ALAPGYYGIQEAAAPTGYQLDGTTYLFQLTSDGQWQYHGTKDNVTSGSVINGQQNLNPVGDKSDDFTVTGDHQQILTLTKYDEPKPSMTLRVIKQDNQSQYLAGAAFTLQPSAGEAETITSSATSGGQAF
Consensus  ALAPGYY.IQEAAAPTGYQLDGTTYLFQLTSDGQWQYHGTKDNVTSGSVINGQQ.LNPVGDKSDDFTVTGDHQQILTLTKYDEPKPSMTLRVIKQDNQ.QYLAGAAFTLQPSAGEAETITSSATS.GQAF

           781     790       800       810       820       830       840       850       860       870       880       890     898
GG         ATKLVADGTYTMSETKAPDGYQSNPAKIAIQVATTGKEATVTIDGEALKPGESKNGYTLAIDGSTITLQAINQPLAILPHTGGQGYQRLLGIALGLISAAFLLLLVVLIKRRVVKQHD
ATCC53103  ATKLVADGTYTMSETKAPDGYQSNPAKIAIQVATTGKEATVTIDGEALKPGESKNGYTLAIDGSTITLQAINQPLAILPHTGGQGYQRLLGIALGLISAAFLLLLVVLIKRRVVKQHD
LMS2-1     ATKLVADGTYTMSETKAPDGYQSNPAKIAIQVATTGKEATVTIDGEALKPGESKNGYTLAIDGSTITLQAINQPLAILPHTGGQGYQRLLGIALGLISAAFLLLLVVLIKRRVVKQHD
E800       ATKLIADGTYTMSETKAPDGYQSNPAKIAIQVATTGKEATVTIDGEALKPGESKNGYTLAIDGSTITLQAINQPLAILPHTGGQGYQRLLGIALGLISAAFLLLLVVLIKRRVVKQHD
Consensus  ATKL!ADGTYTMSETKAPDGYQSNPAKIAIQVATTGKEATVTIDGEALKPGESKNGYTLAIDGSTITLQAINQPLAILPHTGGQGYQRLLGIALGLISAAFLLLLVVLIKRRVVKQHD
```

B

```
           1       10        20        30        40        50        60        70        80        90        100       110       120       130
           |--------+---------+---------+---------+---------+---------+---------+---------+---------+---------+---------+---------+---------|
       GG  MTKSFRPLVILTFCLALLVSLATTTLQQTQAATVPTTVDVVLHKLLFKDTLPTQQANNGTTKPDFSQADVPLNGVTFTVYDVTADFWQLVSKNGGAIEVAQTTLSQDSYQPASSSLIAQVVTAGQGEAYF
ATCC53103  MTKSFRPLVILTFCLALLVSLATTTLQQTQAATVPTTVDVVLHKLLFKDTLPTQQANNGTTKPDFSQADVPLNGVTFTVYDVTADFWQLVSKNGGAIEVAQTTLSQDSYQPASSSLIAQVVTAGQGEAYF
   LMS2-1  MTKSFRPLVILTFCLALLVSLATTTLQQTQAATVPTTVDVVLHKLLFKDTLPTQQANNGTTKPDFLQADVPLNGVTFTVYDVTADFWQLVSKNGGAIEAAQTTLSQDSYQPASSSLIVQVVTAGQGEAYF
     E800  MTKSFRPLVILTFCLALLVSLATTTLQQTQAATVPTTVDVVLHKLLFKDTMPTQQANNGTTKPDFSQADVPLNGVTFTVYDVTADFWQLVSKNGGAIEAAQTTLSQDSYQPASSSLIAQVVTAGQGEAYF
Consensus  MTKSFRPLVILTFCLALLVSLATTTLQQTQAATVPTTVDVVLHKLLFKDT$PTQQANNGTTKPDF.QADVPLNGVTFTVYDVTADFWQLVSKNGGAIE.AQTTLSQDSYQPASSSLI.QVVTAGQGEAYF

           131     140       150       160       170       180       190       200       210       220       230       241
           |--------+---------+---------+---------+---------+---------+---------+---------+---------+---------+---------+|
       GG  GDLPLRQGQHAAVYLFKETAAPKNIEASQNLVVVMSSNLQHGNQSRIDLFPKNKMVSRHTDAPKKVPKKIRQLLPQTGDTVAAWLSVLGLIIFATVLAFNIKNQKINKWER
ATCC53103  GDLPLRQGQHAAVYLFKETAAPKNIEASQNLVVVMSSNLQHGNQSRIDLFPKNKMVSRHTDAPKKVPKKIRQLLPQTGDTVAAWLSVLGLIIFATVLAFNIKNQKINKWER
   LMS2-1  GDLPLRQGQHAAVYLFKETAAPKNIEASQNLVVVMSSNLQHGNQSRIDLFPKNKMVSRHTDAPKKVPKKTRQLLPQTGDTVAAWLSMLGLLIFATVLAFNIKNQKINKWER
     E800  GDLPLRQGQHAAVYLFKETAAPKNIEASQNLVVVMSSNLQHGNQSRIDLFPKNKMVSRHTDVPKKVPKKTRQLLPQTGDTVAAWLSMLGLIIFATVLAFNIKNQKINKWER
Consensus  GDLPLRQGQHAAVYLFKETAAPKNIEASQNLVVVMSSNLQHGNQSRIDLFPKNKMVSRHTD.PKKVPKK.RQLLPQTGDTVAAWLS.LGL.IFATVLAFNIKNQKINKWER
```

C

```
           1       10        20        30        40        50        60        70        80        90       100       110       120       130
           |--------+---------+---------+---------+---------+---------+---------+---------+---------+---------+---------+---------+---------|
       GG  MKKTIAKKVLTLTSTILMTLLMVLGFNGTRVQADTNDTTTQNVVLTKYGFDKDVTAIDRATDQIWTGDGAKPLQGVDFTIYNVTANYWASPKDYKGSFDSAPVAATGTTNDKGQLTQALPIQSKDASGKT
ATCC53103  MKKTIAKKVLTLTSTILMTLLMVLGFNGTRVQADTNDTTTQNVVLTKYGFDKDVTAIDRATDQIWTGDGAKPLQGVDFTIYNVTANYWASPKDYKGSFDSAPVAATGTTNDKGQLTQALPIQSKDASGKT
     E800  MKKTIAKKVLTLTSTILMTLLMVLGFNGTRVQADTNDTTKQNVVLTKYGFDKDVTAIDRATDQMWNGNGAKPLQGVDFTIYNVTANYWASPKDYKGGFDSASVAATGTTNDKGQLTQALPTKSKDASGKT
   LMS2-1  MKKTIAKKVLTLTSTILMTLLMVLGFNGTRVQADTNDTTTQNVVLTKYGFDKDVTAIDRATNQTWDGGGAKRLQGVDFTVYNVTANYWASPKDYKGNFDSAPVAATGTTDDQGELAKALPTQSKDASGKT
Consensus  MKKTIAKKVLTLTSTILMTLLMVLGFNGTRVQADTNDTT.QNVVLTKYGFDKDVTAIDRAT#Q.W.G.GAK.LQGVDFT!YNVTANYWASPKDYKG.FDSA.VAATGTT#D.G#L..ALP..SKDASGKT

           131    140       150       160       170       180       190       200       210       220       230       240       250       260
           |--------+---------+---------+---------+---------+---------+---------+---------+---------+---------+---------+---------+---------|
       GG  RAAVYLFHETNPRAGYNTSADFWLTLPAKAAADGNVYVYPKNVQKTTYERTFVKKDAETKEVLEGAGFKISNSDGKFLKLTDKDGQSVSIGEGFIDVLANNYRLTWVAESDATVFTSDKSGKFGLNGFAD
ATCC53103  RAAVYLFHETNPRAGYNTSADFWLTLPAKAAADGNVYVYPKNVQKTTYERTFVKKDAETKEVLEGAGFKISNSDGKFLKLTDKDGQSVSIGEGFIDVLANNYRLTWVAESDATVFTSDKSGKFGLNGFAD
     E800  RAAVYLFHETNPRAGYNTSADFWLTLPAKAAADGNVYVYPKNVQKTTYERTFVKKDAETKEVLEGAGFKISNSDGKFLKLTDKDGQSVSIGEGFIDVLANNYRLTWVAESDATVFTSDKSGKFGLNGFAD
   LMS2-1  RAAVYLFHETNPRAGYNTSADFWLTLPAKAAADGNVYVYPKNVQKTTYERTFVKKDAETKEVLEGAGFKISNSDGKFLKLTDKDGQSVSIGEGFIDVLANNYRLTWVAESDATVFTSDKSGKFGLNGFAD
Consensus  RAAVYLFHETNPRAGYNTSADFWLTLPAKAAADGNVYVYPKNVQKTTYERTFVKKDAETKEVLEGAGFKISNSDGKFLKLTDKDGQSVSIGEGFIDVLANNYRLTWVAESDATVFTSDKSGKFGLNGFAD

           261    270       280       290       300       310       320       330 334
           |--------+---------+---------+---------+---------+---------+---------+---|
       GG  NTTTYTAVETNVPDGYDAAANTDFKADNSSSDILDAPSGILPHTGGTGTVIFAILGVALIAFGAVAYRKRRNGF
ATCC53103  NTTTYTAVETNVPDGYDAAANTDFKADNSSSDILDAPSGILPHTGGTGTVIFAILGVALIAFGAVAYRKRRNGF
     E800  NTTTYTAVETKVPDGYDAAANTHFKADNSSSDILDAPSGILPHTGGTGTVIFAILGVALIAFGAVAYRKRRNGF
   LMS2-1  NTTNYTAVETNVPDGYDAAANTGFKADNSSSDILDAPSGILPHTGGTGTVIFAILGVALIAFGAVAYRKRRNGF
Consensus  NTT.YTAVET.VPDGYDAAANT.FKADNSSSDILDAPSGILPHTGGTGTVIFAILGVALIAFGAVAYRKRRNGF
```

D

```
           1       10        20        30        40        50        60        70        80        90        100       110       120       130
           |--------+---------+---------+---------+---------+---------+---------+---------+---------+---------+---------+---------+---------|
GG         VTKRTRRPLDLIDIVIGCLLLAGFGVLCYPFASDAYVSYQNQQVIDRYRQQEARKNQMVLRREYNDYQQKNKQLAASQQVPGVASFNHAVNDQGTAKTAAKRNQQILTRQTVAQLTIPKIGLSLPVFDHT
ATCC53103  VTKRTRRPLDLIDIVIGCLLLAGFGVLCYPFASDAYVSYQNQQVIDRYRQQEARKNQMVLRREYNDYQQKNKQLAASQQVPGVASFNHAVNDQGTAKTAAKRNQQILTRQTVAQLTIPKIGLSLPVFDHT
LMS2-1     VTKRTRRPLGLIDIVIGCLLLAGFGVLCYPFASDAYVSYQNQQVIDRYRQQEARKNQMVLRREYNDYQQKNKQLAASQQVPGVASFNHAVNDQGTAKTAAKRNQPILTRQTVAQLTIPKIGLSLPVFDHT
E800       VTKRTRRPLGLIDIVIGCLLLAGFGVLCYPFASDAYVSYQNQQVIDRYRQQEARKNQMVLRREYNDYQQKNKQLAASQQVPGVASFNHAVNDQGTAKTAAKRNQQILTRQTVAQLTIPKIGLSLPVFDHT
Consensus  VTKRTRRPL.LIDIVIGCLLLAGFGVLCYPFASDAYVSYQNQQVIDRYRQQEARKNQMVLRREYNDYQQKNKQLAASQQVPGVASFNHAVNDQGTAKTAAKRNQ.ILTRQTVAQLTIPKIGLSLPVFDHT

           131     140       150       160       170       180       190       200       210       220       230       240       250       260
           |--------+---------+---------+---------+---------+---------+---------+---------+---------+---------+---------+---------+---------|
GG         SDWLLQFGACLLDGTSYPTGGKNTHAVISAHRGVPNAELFTRVPALKKGDKFFISIGNHKLAYQVFKRQVIEPSDTRQLRIVPGQDLVTLMTCTPYMINSHRLLITGRRIPYVKADEEASSWAVWWNKLK
ATCC53103  SDWLLQFGACLLDGTSYPTGGKNTHAVISAHRGVPNAELFTRVPALKKGDKFFISIGNHKLAYQVFKRQVIEPSDTRQLRIVPGQDLVTLMTCTPYMINSHRLLITGRRIPYVKADEEASSWAVWWNKLK
LMS2-1     SDWLLQFGACLLDGTSYPTGGKNTHAVISAHRGVPNAELFTRVPALKKGDKFFISIGNHKLAYQVFKRQVIEPSDTRQLRIVPGQDLVTLMTCTPYMINSHRLLITGRRIPYVKSDDEASSWAVWWNKLK
E800       SDWLLQFGACLLDGTSYPTGGKNTHAVISAHRGVPNAELFNRVPALKKGDKFFISIGNHKLAYQVFKRQVIEPSDTRQLRIVPGQDLVTLMTCTPYMINSHRLLITGRRIPYVKADDEASSWAVWWNKLK
Consensus  SDWLLQFGACLLDGTSYPTGGKNTHAVISAHRGVPNAELF.RVPALKKGDKFFISIGNHKLAYQVFKRQVIEPSDTRQLRIVPGQDLVTLMTCTPYMINSHRLLITGRRIPYVK.D#EASSWAVWWNKLK

           261     270       280       290       300       310       320       330       340       350      359
           |--------+---------+---------+---------+---------+---------+---------+---------+---------+--------|
GG         LIVALLGAVIILGVIGFVMRSLMLGRKHYLLEVPAEATQVVVKRGRHIHSFKSDQTGVTDISLPGNHYRVAIVTPLGRTKYKAYVKKIRDKSFQLKEYH
ATCC53103  LIVALLGAVIILGVIGFVMRSLMLGRKHYLLEVPAEATQVVVKRGRHIHSFKSDQTGVTDISLPGNHYRVAIVTPLGRTKYKAYVKKIRDKSFQLKEYH
LMS2-1     LIVALLGAVIILGVIGFVMRSLMLGRKHYLLEVPAEATQVVVKRGRHIHSFKSDQTGVTDISLPGNHYRVAIVTPLGRTKYKAYVKKIRDKSFQLKEYH
E800       LIVALLGAVIILGVIGFVMRSLMLGRKHYLLEVPAEATQVVVKRGRHIHSFKSDQTGVTDISLPGNHYRVAIVTPLGRTKYKAYVKKIRDKSFQLKEYH
Consensus  LIVALLGAVIILGVIGFVMRSLMLGRKHYLLEVPAEATQVVVKRGRHIHSFKSDQTGVTDISLPGNHYRVAIVTPLGRTKYKAYVKKIRDKSFQLKEYH
```

E

```
           1       10        20        30        40        50        60        70        80        90        100       110       120       130
           |--------+---------+---------+---------+---------+---------+---------+---------+---------+---------+---------+---------+---------|
LC705      LRLFGEEKTRYRLYKSGKLWLVALIGVFALAIGHQPNQVKASSMATRTATLAVQPATLGQELNLNNQQTMNADSPTSSNEVVVKCVDDAGNTLVKDMILQGEVGKVYTIKPATIANYQYTKLANGSAPIN
ATCC8530   LRLFGEEKTRYRLYKSGKLWLVALIGVFALAIGHQPNQVKASSMATRTATLAVQPATLGQELNLNNQQTMNADSPTSSNEVVVKCVDDAGNTLVKDMILQGEVGKVYTIKPATIANYQYTKLANGSAPIN
LMS2-1     LRLFGEEKTRYRLYKSGKLWLVALIGVFALAIGHQPNQVKASSMATRTATLAVQPATLGQELNLNNQQTMNADSPTSSNEVVVKCVDDAGNTLVKDMILQGEVGKVYTIKPATIANYQYTKLANGSAPIN
GG         LRLFGEEKTRYRLYKSGKLWLVALIGVFALAIGHQPSHVKASSVATRTATLAVQPATLGQELNLNNQQTINADSPTSSNEVVVKCVDDAGNTLVKDTVLQGEVGKPYTIKPATIANYQYAKLANGSAPIN
ATCC53103  LRLFGEEKTRYRLYKSGKLWLVALIGVFALAIGHQPSHVKASSVATRTATLAVQPATLGQELNLNNQQTINADSPTSSNEVVVKCVDDAGNTLVKDTVLQGEVGKPYTIKPATIANYQYAKLANGSAPIN
PEL5       LRLFGEEKTRYRLYKSGKLWLVALIGVFALAIGHQPSHVKASSVATRTATLAVQPATLGQELNLNNQQTINADSPTSSNEVVVKCVDDAGNTLVKDTVLQGEVGKPYTIKPATIANYQYAKLANGSAPIN
PEL6       LRLFGEEKTRYRLYKSGKLWLVALIGVFALAIGHQPSHVKASSVATRTATLAVQPATLGQELNLNNQQTINADSPTSSNEVVVKCVDDAGNTLVKDTVLQGEVGKPYTIKPATIANYQYAKLANGSAPIN
ATCC21052  LRLFGEEKTRYRLYKSGKLWLVALIGVFALAIGHQPSHVKASSVATRTATLAVQPATLGQELNLNNQQTINADSPTSSNEVVVKCVDDAGNTLVKDTVLQGEVGKPYTIKPATIANYQYAKLANGSAPIN
R0011      LRLFGEEKTRYRLYKSGKLWLVALIGVFALAIGHQPSHVKASSVATRTATLAVQPATLGQELNLNNQQTINADSPTSSNEVVVKCVDDAGNTLVKDTVLQGEVGKPYTIKPATIANYQYAKLANGSAPIN
LRHMDP2    LRLFGEEKTRYRLYKSGKLWLVALIGVFALAIGHQPSHVKASSMATRTATLAVQPATLGQKLNLNNQQTINADSPTSSNEVVVKCVDDAGNTLVKDTVLQGEVGKPYTIKPATIANYQYAKLANGSAPIN
LRHMDP3    LRLFGEEKTRYRLYKSGKLWLVALIGVFALAIGHQPSHVKASSMATRTATLAVQPATLGQKLNLNNQQTINADSPTSSNEVVVKCVDDAGNTLVKDTVLQGEVGKPYTIKPATIANYQYAKLANGSAPIN
HN001      LRLFGEEKTRYRLYKSGKLWLVALIGFFVLAIGHQPNQVKASSMATRTAILAVQPATLGQELNLNNQQTINADSPTSSNEVVVKCVDDAGNTLVKDTVLQGEVGKPYTIKPATIANYQYAKLANGSAPIN
E800       LRLFGEEKTRYRLYKSGKLWLVALIGFFVLAIGHQPNQVKASSMATRTAILAVQPATLGQELNLNNQQTINADSPTSSNEVVVKCVDDAGNTLVKDTVLQGEVGKPYTIKPATIANYQYAKLANGSAPIN
Consensus  LRLFGEEKTRYRLYKSGKLWLVALIG.F.LAIGHQP..VKASS.ATRTA.LAVQPATLGQ.LNLNNQQT.NADSPTSSNEVVVKCVDDAGNTLVKD.!LQGEVGK.YTIKPATIANYQY.KLANGSAPIN

           131     140       150       160       170       180       190       200       210       220       230       240       250       260
           |--------+---------+---------+---------+---------+---------+---------+---------+---------+---------+---------+---------+---------|
LC705      GTFSKGTLTVTLVYTKVPVAQRTVNVKYVDEHGNEIAPATTLIGTVGGSYTAVPANVKNYEYAHLAANSAPEKGSFTANPQTVTFVYTEKPAAQGSVTERFVDEAGKRIAPDKTLTGQVGDLYEARPIEI
ATCC8530   GTFSKGTLTVTLVYTKVPVAQRTVNVKYVDEHGNEIAPATTLIGTVGGSYTAVPANVKNYEYAHLAANSAPEKGSFTANPQTVTFVYTEKPAAQGSVTERFVDEAGKRIAPDKTLTGQVGDLYEARPIEI
LMS2-1     GTFSKGTLTVTLVYTKVPVAQRTVNVKYVDEHGNEIAPATTLIGTVGGSYTAVPANVKNYEYAHLAANSAPEKGSFTANPQTVTFVYTEKPAAQGSVTERFVDEAGKRIAPDKTLTGQVGDLYEARPIEI
GG         GTFSKGTLTVTLVYTKVPVTQRTVNVKYVDEHGNEIAPATTLTGTVGGSYTAVPANVKNYEYAHLAANSAPEKGSFTANPQTVTFVYTEKPAAQGSVTERFVDEAGKRIAPDKTLTGQVGDLYEARPIEI
ATCC53103  GTFSKGTLTVTLVYTKVPVTQRTVNVKYVDEHGNEIAPATTLTGTVGGSYTAVPANVKNYEYAHLAANSAPEKGSFTANPQTVTFVYTEKPAAQGSVTERFVDEAGKRIAPDKTLTGQVGDLYEARPIEI
PEL5       GTFSKGTLTVTLVYTKVPVTQRTVNVKYVDEHGNEIAPATTLTGTVGGSYTAVPANVKNYEYAHLAANSAPEKGSFTANPQTVTFVYTEKPAAQGSVTERFVDEAGKRIAPDKTLTGQVGDLYEARPIEI
PEL6       GTFSKGTLTVTLVYTKVPVTQRTVNVKYVDEHGNEIAPATTLTGTVGGSYTAVPANVKNYEYAHLAANSAPEKGSFTANPQTVTFVYTEKPAAQGSVTERFVDEAGKRIAPDKTLTGQVGDLYEARPIEI
ATCC21052  GTFSKGTLTVTLVYTKVPVTQRTVNVKYVDEHGNEIAPATTLTGTVGGSYTAVPANVKNYEYAHLAANSAPEKGSFTANPQTVTFVYTEKPAAQGSVTERFVDEAGKRIAPDKTLTGQVGDLYEARPIEI
R0011      GTFSKGTLTVTLVYTKVPVTQRTVNVKYVDEHGNEIAPATTLTGTVGGSYTAVPANVKNYEYAHLAANSAPEKGSFTANPQTVTFVYTEKPAAQGSVTERFVDEAGKRIAPDKTLTGQVGDLYEARPIEI
LRHMDP2    GTFSKGTLTVTLVYTKVPVAQRTVNVKYVDEHGNEIAPAITLTGTVGGSYTAVPANVKNYEYAHLAANSAPEKGSFTANPQTVTFVYTEKPAAQGSVTERFVDEAGKRIAPDKTLTGQVGDLYEARPIDI
LRHMDP3    GTFSKGTLTVTLVYTKVPVAQRTVNVKYVDEHGNEIAPATTLTGTVGGSYTAVPANVKNYEYAHLAANSAPEKGSFTANPQTVTFVYTEKPAAQGSVTERFVDEAGKRIAPDKTLTGQVGDLYEARPIDI
HN001      GTFSKGTLTVTLVYTKVPVAQRTVNVKYVDEHGSEIAPATTLTGTVGGSYTAVPANVKNYEYVHLAANSAPEKGSFTANPQTVTFVYTEKPAAQGSVTERFVDEAGKRIAPDKMLTGQVGDLYEARPIEI
E800       GTFSKGTLTVTLVYTKVPVAQRTVNVKYVDEHGSEIAPATTLTGTVGGSYTAVPANVKNYEYVHLAANSAPEKGSFTANPQTVTFVYTEKPAAQGSVTERFVDEAGKRIAPDKMLTGQVGDLYEARPIEI
Consensus  GTFSKGTLTVTLVYTKVPV.QRTVNVKYVDEHG.EIAPA.TL.GTVGGSYTAVPANVKNYEY.HLAANSAPEKGSFTANPQTVTFVYTEKPAAQGSVTERFVDEAGKRIAPDK.LTGQVGDLYEARPI#I

           261     270       280       290       300       310       320       330       340       350       360       370       380       390
           |--------+---------+---------+---------+---------+---------+---------+---------+---------+---------+---------+---------+---------|
LC705      SDYAFSRVAQGSAPAGNAFINGNVIVTFVYKQVPATQGSVTVRYVDENGNELAPNKVLTGQSGSAYTTGPITINGYRYVRLAADSAAASGTFPKDTGLVVSFVYTKPAIPVTPTTPETSTVPSTSSQSAT
ATCC8530   SDYAFSRVAQGSAPAGNAFINGNVIVTFVYKQVPATQGSVTVRYVDENGNELAPNKVLTGQSGSAYTTGPITINGYRYVRLAADSAAASGTFPKDTGLVVSFVYTKPAIPVTPTTPETSTVPSTSSQSAT
LMS2-1     SDYAFSRVAQGSAPAGNAFINGNVIVTFVYKQVPATQGSVTVRYVDENGNELAPNKVLTGQSGSAYTTGPITINGYRYVRLAADSAAASGTFPKDTGLVVSFVYTKPAIPVTPTTPETSTVPSTSSQSAT
GG         SDYAFSRVAQGSAPAGNTFINGNVIVTFVYKQVPATQGSVTVRYVDENGNELAPNRVLAGQSGSAYTTGPITINGYRYVRLAADSAAASGTFPKDTGLVVSFVYTKPAIPVTPTTPETSTVPSTSSQSAT
ATCC53103  SDYAFSRVAQGSAPAGNTFINGNVIVTFVYKQVPATQGSVTVRYVDENGNELAPNRVLAGQSGSAYTTGPITINGYRYVRLAADSAAASGTFPKDTGLVVSFVYTKPAIPVTPTTPETSTVPSTSSQSAT
PEL5       SDYAFSRVAQGSAPAGNTFINGNVIVTFVYKQVPATQGSVTVRYVDENGNELAPNRVLAGQSGSAYTTGPITINGYRYVRLAADSAAASGTFPKDTGLVVSFVYTKPAIPVTPTTPETSTVPSTSSQSAT
PEL6       SDYAFSRVAQGSAPAGNTFINGNVIVTFVYKQVPATQGSVTVRYVDENGNELAPNRVLAGQSGSAYTTGPITINGYRYVRLAADSAAASGTFPKDTGLVVSFVYTKPAIPVTPTTPETSTVPSTSSQSAT
ATCC21052  SDYAFSRVAQGSAPAGNTFINGNVIVTFVYKQVPATQGSVTVRYVDENGNELAPNKVLTGQSGSAYTTGPITINGYRYVRLAADSAAASGTFPKDTGLVVSFVYTKPAIPVMPTMPETSTVPSTSSQSAT
R0011      SDYAFSRVAQGSAPAGNTFINGNVIVTFVYKQVPATQGSVTVRYVDENGNELAPNKVLTGQSGSAYTTGPITINGYRYVRLAADSAAASGTFPKDTGLVVSFVYTKPAIPVMPTMPETSTVPSTSSQSAT
LRHMDP2    SDYAFSRVAQGSAPAGNTFINGNVIVTFVYKQVPATQGSVTVRYVDENGNELAPNKVLTGQSGSAYTTGPITINGYRYVRLAADSAAASGTFPKDTGLVVSFVYTKPVIPVTPTMPETSTVPSTSSQSAT
LRHMDP3    SDYAFSRVAQGSAPAGNTFINGNVIVTFVYKQVPATQGSVTVRYVDENGNELAPNKVLTGQSGSAYTTGPITINGYRYVRLAADSAAASGTFPKDTGLVVSFVYTKPVIPVTPTMPETSTVPSTSSQSAT
HN001      SDYAFSCVAQGSAPAGNTFINGNVIVTFVYKQVPATQGSVTVRYVDENGNELAPNKVLTGQSGSAYTTGPITINGYRYVRLAADSAAASGTFPKDTGLVVSFVYTKPAIPVTSTTPETSTVPSTSSQSAT
E800       SDYAFSCVAQGSAPAGNTFINGNVIVTFVYKQVPATQGSVTVRYVDENGNELAPNKVLTGQSGSAYTTGPITINGYRYVRLAADSAAASGTFPKDTGLVVSFVYTKPAIPVTSTTPETSTVPSTSSQSAT
Consensus  SDYAFS.VAQGSAPAGN.FINGNVIVTFVYKQVPATQGSVTVRYVDENGNELAPN.VL.GQSGSAYTTGPITINGYRYVRLAADSAAASGTFPKDTGLVVSFVYTKP.IPV..T.PETSTVPSTSSQSAT

           391     400       410       420       430     438
           |--------+---------+---------+---------+-------|
LC705      TEVITPSAQRRLPNTNEKHEYGIAAVGLALLSLMGLGSTQLFRKAKRQ
ATCC8530   TEVITPSAQRRLPNTNEKHEYGIAAVGLALLSLMGLGSTQLFRKAKRQ
LMS2-1     TEVITPSAQRRLPNTNEKHEYGIAAVGLALLSLMGLGSTQLFRKAKRQ
GG         TEVITPSAQRRLPNTNEKHEYGIAAVGLALLSLMGLGSTLLFRKAKRQ
ATCC53103  TEVITPSAQRRLPNTNEKHEYGIAAVGLALLSLMGLGSTLLFRKAKRQ
PEL5       TEVITPSAQRRLPNTNEKHEYGIAAVGLALLSLMGLGSTLLFRKAKRQ
PEL6       TEVITPSAQRRLPNTNEKHEYGIAAVGLALLSLMGLGSTLLFRKAKRQ
ATCC21052  TEVITPSAQRRLPNTNEKHEHGIAAVGLALLSLMGLGSTQLFRKAKRQ
R0011      TEVITPSAQRRLPNTNEKHEHGIAAVGLALLSLMGLGSTQLFRKAKRQ
LRHMDP2    TEVITPSAQRRLPNTNEKHEYGIAAVGLALLSLMGLGSTQLFRKAKRQ
LRHMDP3    TEVITPSAQRRLPNTNEKHEYGIAAVGLALLSLMGLGSTQLFRKAKRQ
HN001      TEVITPSTQRRLPNTNEKHEYGIAAVGLALLSLMGFGSTQLFRKAKRQ
E800       TEVITPSTQRRLPNTNEKHEYGIAAVGLALLSLMGFGSTQLFRKAKRQ
Consensus  TEVITPS.QRRLPNTNEKHE.GIAAVGLALLSLMG.GST.LFRKAKRQ
```

F

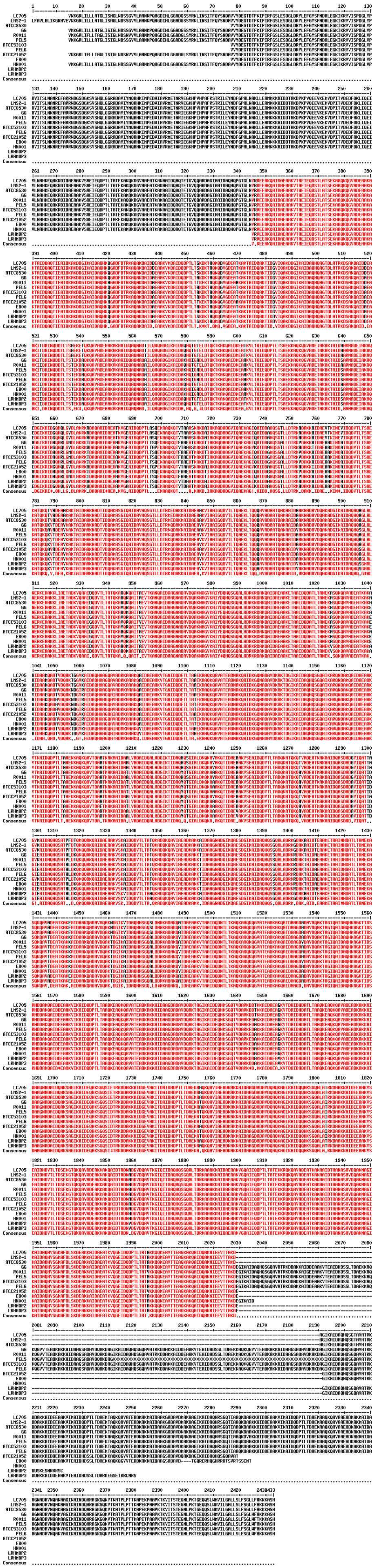

G

```
            1        10        20        30        40        50        60        70        80        90        100       110       120       130
LC705       MVDSKKVLSVTAGFVGAAGLAALATGANTVSASTGTVSYKSGATTVWNSPSWHQVKRYVTFGDTVQLLGKTVDQNGATWYKVGDNQWIPELYLNVAGKTATVETPSSAASQTAVSQAPASQAPTSQAPAT
ATCC8530    MVDSKKVLSVTAGFVGAAGLAALATGANTVSASTGTVSYKSGATTVWNSPSWHQVKRYVTFGDTVQLLGKTVDQNGATWYKVGDNQWIPELYLNVAGKTATVETPSSAASQTAVSQAPASQAPTSQAPAT
LMS2-1      MVDSKKVLSVTAGFVGAAGLAALATGANTVSASTGTVSYKSGATTVWNSPSWHQVKRYVTFGDTVQLLGKTVDQNGATWYKVGDNQWIPELYLNVAGKTATVETPSSAASQTAVSQAPASQAPTSQAPAT
GG          MVDSKKVLSVTAGFVGAAGLAALATGANTVSASTGTVSYKSGATTVWNSPSWHQVKRYVTFGDTVQLLGKTVDQNGATWYKVGDNQWIPELYLNVAGKTATVETPSSAASQTAVSQAPASQAPTSQAPAT
ATCC53103   MVDSKKVLSVTAGFVGAAGLAALATGANTVSASTGTVSYKSGATTVWNSPSWHQVKRYVTFGDTVQLLGKTVDQNGATWYKVGDNQWIPELYLNVAGKTATVETPSSAASQTAVSQAPASQAPTSQAPAT
PEL6        MVDSKKVLSVTAGFVGAAGLAALATGANTVSASTGTVSYKSGATTVWNSPSWHQVKRYVTFGDTVQLLGKTVDQNGATWYKVGDNQWIPELYLNVAGKTATVETPSSAASQTAVSQAPASQAPTSQAPAT
PEL5        MVDSKKVLSVTAGFVGAAGLAALATGANTVSASTGTVSYKSGATTVWNSPSWHQVKRYVTFGDTVQLLGKTVDQNGATWYKVGDNQWIPELYLNVAGKTATVETPSSAASQTAVSQAPASQAPTSQAPAT
E800        MVDSKKVLSVTAGFVGAAGLAALATGANTVSASTGTVSYKSGATTVWNSPSWHQVKRYVTFGDTVQLLGKTVDQNGATWYKVGDNQWIPELYLNVAGKTATVETPSSAASQTAVSQAPASQAPTSQAPAT
R0011       MVDSKKVLSVTAGFVGAAGLAALATGANTVSASTGTVSYKSGATTVWNSPSWHQVKRYVTFGDTVQLLGKTVDQNGATWYKVGDNQWIPELYLNVAGKTATVETPSSAASQTAVSQAPASQAPTSQAPAT
HN001       MVDSKKVLSVTAGFVGAAGLAALATGANTVSASTGTVSYKSGATTVWNSPSWHQVKRYVTFGDTVQLLGKTVDQNGATWYKVGDNQWIPELYLNVAGKTATVETPSSAASQTAVSQAPASQAPTSQAPAT
ATCC21052   MVDSKKVLSVTAGFVGAAGLAALATGANTVSASTGTVSYKSGATTVWNSPSGHQVKRYVTFGDTVQLLGKTVDQNGATWYKVGDNQWIPELYLNVAGKTATVETPSSAASQTAVSQAPASQAPTSQAPAT
LRHMDP2     MVDSKKVLSVTAGFVGAAGLAALATGANTVSASTGTVSYKSGATTVWNSPSWHQVKRYVTFGDTVQLLGKTVDQNGATWYKVGDNQWIPELYLNVAGKTATVETPSSAASQTAVSQAPASQAPTSQAPAT
LRHMDP3     MVDSKKVLSVTAGFVGAAGLAALATGANTVSASTGTVSYKSGATTVWNSPSWHQVKRYVTFGDTVQLLGKTVDQNGATWYKVGDNQWIPELYLNVAGKTATVETPSSAASQTAVSQAPASQAPTSQAPAT
Consensus   MVDSKKVLSVTAGFVGAAGLAALATGANTVSASTGTVSYKSGATTVWNSPS.HQVKRYVTFGDTVQLLGKTVDQNGATWYKVGDNQWIPELYLNVAGKTATVETPSSAASQTAVSQAPASQAPTSQAPAT

            131 140       150       160       170       180       190       200       210       220       230       240       250       260
LC705       QTPAAPQTDTQTANTQLYVKNIGSAVTVWTTPAYTHATGQYLEGSQTLTAVAQQQANGETWYRLANGGYVPARFVSTTPVAVTPQPAAPQSNEASVASTNTNAANDSAAASSAAASQAAASSAAASTAAA
ATCC8530    QTPAAPQTDTQTANTQLYVKNIGSAVTVWTTPAYTHATGQYLEGSQTLTAVAQQQANGETWYRLANGGYVPARFVSTTPVAVTPQPAAPQSNEASVASTNTNAANDSAAASSAAASQAAASSAAASTAAA
LMS2-1      QTPAAPQTDTQTANTQLYVKNIGSAVTVWTTPAYTHATGQYLEGSQTLTAVAQQQANGETWYRLANGGYVPARFVSTTPVAVTPQPAAPQSNEASVASTNTNAANDSAAASSAAASQAAASSAAASTAAA
GG          QTPAAPQTDTQTANTQLYVKNIGSAVTVWTTPAYTHATGQYLEGSQTLTAVAQQQANGETWYRLANGGYVPARFVSTTPVAVTPQPAAPQSNEASVASTNTNAANDSAAASSAAASQAAASSAAASTAAA
ATCC53103   QTPAAPQTDTQTANTQLYVKNIGSAVTVWTTPAYTHATGQYLEGSQTLTAVAQQQANGETWYRLANGGYVPARFVSTTPVAVTPQPAAPQSNEASVASTNTNAANDSAAASSAAASQAAASSAAASTAAA
PEL6        QTPAAPQTDTQTANTQLYVKNIGSAVTVWTTPAYTHATGQYLEGSQTLTAVAQQQANGETWYRLANGGYVPARFVSTTPVAVTPQPAAPQSNEASVASTNTNAANDSAAASSAAASQAAASSAAASTAAA
PEL5        QTPAAPQTDTQTANTQLYVKNIGSAVTVWTTPAYTHATGQYLEGSQTLTAVAQQQANGETWYRLANGGYVPARFVSTTPVAVTPQPAAPQSNEASVASTNTNAANDSAAASSAAASQAAASSAAASTAAA
E800        QTPAAPQTDTQTANTQLYVKNIGSAVTVWTTPAYTHATGQYLEGSQTLTAVAQQQANGETWYRLANGGYVPARFVSTTPVAVTPQPAAPQSNEASVASTNTNAANDSAAASSAAASQAAASSAAASTAAA
R0011       QTPAAPQTDTQTANTQLYVKNIGSAVTVWTTPAYTHATGQYLEGSQTLTAVAQQQANGETWYRLANGGYVPARFVSTTPVAVTPQPAAPQSNEASVASTNTNAANDSAAASSAAASQAAASSAAASTAAA
HN001       QTPAAPQTDTQTANTQLYVKNIGSAVTVWTTPAYTHATGQYLEGSQTLTAVAQQQANGETWYRLANGGYVPARFVSTTPVAVTPQPAAPQSNEASVASTNTNAANDSAAASSAAASQAAASSAAASTAAA
ATCC21052   QTPAAPQTDTQTANTQLYVKNIGSAVTVWTTPAYTHATGQYLEGSQTLTAVAQQQANGETWYRLANGGYVPARFVSTTPVAVTPQPAAPQSNEASVASTNTNAANDSAAASSAAASQAAASSAAASTAAA
LRHMDP2     QTPAAPQTDTQTANTQLYVKNIGSAVTVWTTPAYTHATGQYLEGSQTLTAVAQQQANGETWYRLANGGYVPARFVSTTPVAVTPQPAAPQSNEASVASTNTNAANDSAAASSAAASQAAASSAAASTAAA
LRHMDP3     QTPAAPQTDTQTANTQLYVKNIGSAVTVWTTPAYTHATGQYLEGSQTLTAVAQQQANGETWYRLANGGYVPARFVSTTPVAVTPQPAAPQSNEASVASTNTNAANDSAAASSAAASQAAASSAAASTAAA
Consensus   QTPAAPQTDTQTANTQLYVKNIGSAVTVWTTPAYTHATGQYLEGSQTLTAVAQQQANGETWYRLANGGYVPARFVSTTPVAVTPQPAAPQSNEASVASTNTNAANDSAAASSAAASQAAASSAAASTAAA

            261 270       280       290       300       310       320       330       340       350       360       370       380       390
LC705       NAAVASANAIASQAAASEAAASQAAASQAAASQAAASQAAASQAAASQAAASQAAA-----NAAQQAPANQANVTTTQVNANQAQQQTATATPAVNTSNQTAAVSASRQAKIQAVIAIAE
ATCC8530    NAAVASANAIASQAAASEAAASQAAASQAAASQAAASQAAASQAAASQAAASQAAA-----NAAQQAPANQANVTTTQVNANQAQQQTATATPAVNTSNQTAAVSASRQAKIQAVIAIAE
LMS2-1      NAAVASANAIASQAAASEAAASQAAASQAAASQAAASQAAASQAAASQAAASQAAA-----NAAQQAPANQANVTTTQVNANQAQQQTATATPAVNTSNQTAAVSASRQAKIQAVIAIAE
GG          NAAVASANATASQAAASEAAASQAAASQAAASQAAASQAAASQAAASQAAASQAAASQAAANAAQQAPANQANVTTTQVNANQAQQQTATATPAVNTSNQTAAVSASRQAKIQAVIAIAE
ATCC53103   NAAVASANATASQAAASEAAASQAAASQAAASQAAASQAAASQAAASQAAASQAAASQAAANAAQQAPANQANVTTTQVNANQAQQQTATATPAVNTSNQTAAVSASRQAKIQAVIAIAE
PEL6        NAAVASANATASQAAASEAAASQAAASQAAASQAAASQAAASQAAASQAAASQAAASQAAANAAQQAPANQANVTTTQVNANQAQQQTATATPAVNTSNQTAAVSASRQAKIQAVIAIAE
PEL5        NAAVASANATASQAAASEAAASQAAASQAAASQAAASQAAASQAAASQAAASQAAASQAAANAAQQAPANQANVTTTQVNANQAQQQTATATPAVNTSNQTAAVSASRQAKIQAVIAIAE
E800        NAAVASANATASQAAASEAAASQAAASQAAASQAAASQAAASQAAASQAAASQAAASQAAANAAQQAPANQANVTTTQVNANQAQQQTATATPAVNTSNQTAAVSASRQAKIQAVIAIAE
R0011       NAAVASANATASQAAASEAAASQAAASQAAASQAAASQAAASQAAASQAAASQAAASQAAANAAQQAPANQANVTTTQVNANQAQQQTATATPAVNTSNQTAAVSASRQAKIQAVIAIAE
HN001       NAAVASANATASQAAASEAAASQAAASQAAASQAAASQAAASQAAASQAAASQAAASQAAANAAQQAPANQANVTTTQVNANQAQQQTATATPAVNTSNQTAAVSASRQAKIQAVIAIAE
ATCC21052   NAAVASANATASQAAASEAAASQAAASQAAASQAAASQAAASQAAASQAAASQAAASQAAANAAQQAPANQANVTTTQVNANQAQQQTATATPAVNTSNQTAAVSASRQAKIQAVIAIAE
LRHMDP2     NAAVASANATASQAAASQAAASQAAASQAAASQAAASQAAASQAAA---------------NAAQQAPANQANVTTTQVNANQAQQQTATATPAVNTSNQTAAVSASRQAKIQAVIAIAE
LRHMDP3     NAAVASANATASQAAASQAAASQAAASQAAASQAAASQAAASQAAA---------------NAAQQAPANQANVTTTQVNANQAQQQTATATPAVNTSNQTAAVSASRQAKIQAVIAIAE
Consensus   NAAVASANA.ASQAAAS#AAASQAAASQAAASQAAASQAAASQAAA...............NAAQQAPANQANVTTTQVNANQAQQQTATATPAVNTSNQTAAVSASRQAKIQAVIAIAE

            391 400       410       420       430       440       450       460       470       480       490     498
LC705       QQVGKPYVWGGKGPNSFDCSGLMYYAFLNGAGVNIGGWTVPQESSGTQVSLSALQPGDLLFWGSHGSTYHVALYIGGGTMIQAPQPGENVKYTALAYFMPDFAVRPSL
ATCC8530    QQVGKPYVWGGKGPNSFDCSGLMYYAFLNGAGVNIGGWTVPQESSGTQVSLSALQPGDLLFWGSHGSTYHVALYIGGGTMIQAPQPGENVKYTALAYFMPDFAVRPSL
LMS2-1      QQVGKPYVWGGKGPNSFDCSGLMYYAFLNGAGVNIGGWTVPQESSGTQVSLSALQPGDLLFWGSHGSTYHVALYIGGGTMIQAPQPGENVKYTALAYFMPDFAVRPSL
GG          QQVGKPYVWGGKGPNSFDCSGLMYYAFLNGAGVNIGGWTVPQESSGTQVSLSALQPGDLLFWGSHGSTYHVALYIGGGTMIQAPQPGENVKYTALAYFMPDFAVRPSL
ATCC53103   QQVGKPYVWGGKGPNSFDCSGLMYYAFLNGAGVNIGGWTVPQESSGTQVSLSALQPGDLLFWGSHGSTYHVALYIGGGTMIQAPQPGENVKYTALAYFMPDFAVRPSL
PEL6        QQVGKPYVWGGKGPNSFDCSGLMYYAFLNGAGVNIGGWTVPQESSGTQVSLSALQPGDLLFWGSHGSTYHVALYIGGGTMIQAPQPGENVKYTALAYFMPDFAVRPSL
PEL5        QQVGKPYVWGGKGPNSFDCSGLMYYAFLNGAGVNIGGWTVPQESSGTQVSLSALQPGDLLFWGSHGSTYHVALYIGGGTMIQAPQPGENVKYTALAYFMPDFAVRPSL
E800        QQVGKPYVWGGKGPNSFDCSGLMYYAFLNGAGVNIGGWTVPQESSGTQVSLSALQPGDLLFWGSHGSTYHVALYIGGGTMIQAPQPGENVKYTALAYFMPDFAVRPSL
R0011       QQVGKPYVWGGKGPNSFDCSGLMYYAFLNGAGVNIGGWTVPQESSGTQVSLSALQPGDLLFWGSHGSTYHVALYIGGGTMIQAPQPGENVKYTALAYFMPDFAVRPSL
HN001       QQVGKPYVWGGKGPNSFDCSGLMYYAFLNGAGVNIGGWTVPQESSGTQVSLSALQPGDLLFWGSHGSTYHVALYIGGGTMIQAPQPGENVKYTALAYFMPDFAVRPSL
ATCC21052   QQVGKPYVWGGKGPNSFDCSGLMYYAFLNGAGVNIGGWTVPQESSGTQVSLSALQPGDLLFWGSHGSTYHVALYIGGGTMIQAPQPGENVKYTALAYFMPDFAVRPSL
LRHMDP2     QQVGKPYVWGGKGPNSFDCSGLMYYAFLNGAGVNIGGWTVPQESSGTQVSLSALQPGDLLFWGGHGATYHVALYIGGGTMIQAPQPGQNVKYTALAYFMPDFAVRPSL
LRHMDP3     QQVGKPYVWGGKGPNSFDCSGLMYYAFLNGAGVNIGGWTVPQESSGTQVSLSALQPGDLLFWGGHGATYHVALYIGGGTMIQAPQPGQNVKYTALAYFMPDFAVRPSL
Consensus   QQVGKPYVWGGKGPNSFDCSGLMYYAFLNGAGVNIGGWTVPQESSGTQVSLSALQPGDLLFWG.HG.TYHVALYIGGGTMIQAPQPG#NVKYTALAYFMPDFAVRPSL
```

H

```
           1       10        20        30        40        50        60        70        80        90        100       110       120       130
           |--------+---------+---------+---------+---------+---------+---------+---------+---------+---------+---------+---------+---------|
    LC705  MKFNKAMMTLVAAVTLAGSVSAVTPVFADTSASIASNKSETNDLLKQIEAANTEVINLNKQIDAKNGEISDATAKISATDAKIASLSGEITAAQKNVAARKNNLKDQLISLQKKAGSSVSGNVYIDFVLN
 ATCC8530  MKFNKAMMTLVAAVTLAGSVSAVTPVFADTSASIASNKSETNDLLKQIEAANTEVINLNKQIDAKNGEISDATAKISATDAKIASLSGEITAAQKNVAARKNNLKDQLISLQKKAGSSVSGNVYIDFVLN
    R0011  MKFNKAMMTLVAAVTLAGSVSAVTPVFADTSASIASNKSETNDLLKQIEAANTEVINLNKQIDAKNGEISDATAKISATDAKIASLSGEITAAQKNVAARKNNLKDQLISLQKKAGSSVSGNVYIDFVLN
   LMS2-1  MKFNKAMMTLVAAVTLAGSVSAVTPVFADTSASIASNKSETNDLLKQIEAANTEVINLNKQIDAKNGEISDATAKISATDAKIASLSGEITAAQKNVAARKNNLKDQLISLQKKAGSSVSGNVYIDFVLN
ATCC21052  MKFNKAMMTLVAAVTLAGSVSAVTPVFADTSASIASNKSETNDLLKQIEAANTEVINLNKQIDAKNGEISDATAKISATDAKIASLSGEITAAQKNVAARKNNLKDQLISLQKKAGSSVSGNVYIDFVLN
       GG  MKFNKAMMTLVAAVTLAGSVSALTPVFADTSASIASNKSETNDLLKQIEAANTEVINLNKQIDAKNGEISDATAKISATDAKIASLSGEITAAQKNVAARKNNLKDQLISLQKKAGSSVSGNVYIDFVLN
     E800  MKFNKAMMTLVAAVTLAGSVSALTPVFADTSASIASNKSETNDLLKQIEAANTEVINLNKQIDAKNGEISDATAKISATDAKIASLSGEITAAQKNVAARKNNLKDQLISLQKKAGSSVSGNVYIDFVLN
     PEL5  MKFNKAMMTLVAAVTLAGSVSALTPVFADTSASIASNKSETNDLLKQIEAANTEVINLNKQIDAKNGEISDATAKISATDAKIASLSGEITAAQKNVAARKNNLKDQLISLQKKAGSSVSGNVYIDFVLN
     PEL6  MKFNKAMMTLVAAVTLAGSVSALTPVFADTSASIASNKSETNDLLKQIEAANTEVINLNKQIDAKNGEISDATAKISATDAKIASLSGEITAAQKNVAARKNNLKDQLISLQKKAGSSVSGNVYIDFVLN
ATCC53103  MKFNKAMMTLVAAVTLAGSVSALTPVFADTSASIASNKSETNDLLKQIEAANTEVINLNKQIDAKNGEISDATAKISATDAKIASLSGEITAAQKNVAARKNNLKDQLISLQKKAGSSVSGNVYIDFVLN
    HN001  MKFNKAMMTLVAAVTLAGSVSAVTPVFADTSASIASNKSETNDLLKQIEAANTEVINLNKQIDAKNGEISDATAKISATDAKIASLSGEITAAQKNVAARKNNLKDQLISLQKKAGSSVSGNVYIDFVLN
  LRHMDP2  MKFNKAMMTLVAAVTLAGSVSAVTPVFADTSASIASNKSETNDLLKQIEAANTEVINLNKQIDAKNGEISDATAKISATDAKIASLSGEITAAQKNVAARKNNLKDQLISLQKKAGSSVSGNVYIDFVLN
  LRHMDP3  MKFNKAMMTLVAAVTLAGSVSAVTPVFADTSASIASNKSETNDLLKQIEAANTEVINLNKQIDAKNGEISDATAKISATDAKIASLSGEITAAQKNVAARKNNLKDQLISLQKKAGSSVSGNVYIDFVLN
Consensus  MKFNKAMMTLVAAVTLAGSVSA.TPVFADTSASIASNKSETNDLLKQIEAANTEVINLNKQIDAKNGEISDATAKISATDAKIASLSGEITAAQKNVAARKNNLKDQLISLQKKAGSSVSGNVYIDFVLN

           131     140       150       160       170       180       190       200       210       220       230       240       250       260
           |--------+---------+---------+---------+---------+---------+---------+---------+---------+---------+---------+---------+---------|
    LC705  SQSLSDLIARTMTVGKLSQASKDALDAVTVAKDKLAALKSEQETARQTLVSTKASLETQKSQLETLQKTASDKQDALNKEIADHKDELVALQSQFAQEQSEAAKATQAALKTAAASTASSSTSSTSNKSA
 ATCC8530  SQSLSDLIARTMTVGKLSQASKDALDAVTVAKDKLAALKSEQETARQTLVSTKASLETQKSQLETLQKTASDKQDALNKEIADHKDELVALQSQFAQEQSEAAKATQAALKTAAASTASSSTSSTSNKSA
    R0011  SQSLSDLIARTMTVGKLSQASKDALDAVTVAKDKLAALKSEQETARQTLVSTKASLETQKSQLETLQKTASDKQDALNKEIADHKDELVALQSQFAQEQSEAAKATQAALKTAAASTASSSTSSTSNKSA
   LMS2-1  SQSLSDLIARTMTVGKLSQASKDALDAVTVAKDKLAALKSEQETARQTLVSTKASLETQKSQLETLQKTASDKQDALNKEIADHKDELVALQSQFAQEQSEAAKATQAALKTAAASTASSSTSSTSNKSA
ATCC21052  SQSLSDLIARTMTVGKLSQASKDALDAVTVAKDKLAALKSEQETARQTLVSTKASLETQKSQLETLQKTASDKQDALNKEIADHKDELVALQSQFAQEQSEAAKATQAALKTAAASTASSSTSSTSNKSA
       GG  SQSLSDLIARTMTVGKLSQASKDALDAVTVAKDKLAALKSEQETARQTLVSTKASLETQKSQLETLQKTASDKQDALNKEIADHKDELVALQSQFAQEQSEAAKATQAALKTAAASTASSSTSSTSNKSA
     E800  SQSLSDLIARTMTVGKLSQASKDALDAVTVAKDKLAALKSEQETARQTLVSTKASLETQKSQLETLQKTASDKQDALNKEIADHKDELVALQSQFAQEQSEAAKATQAALKTAAASTASSSTSSTSNKSA
     PEL5  SQSLSDLIARTMTVGKLSQASKDALDAVTVAKDKLAALKSEQETARQTLVSTKASLETQKSQLETLQKTASDKQDALNKEIADHKDELVALQSQFAQEQSEAAKATQAALKTAAASTASSSTSSTSNKSA
     PEL6  SQSLSDLIARTMTVGKLSQASKDALDAVTVAKDKLAALKSEQETARQTLVSTKASLETQKSQLETLQKTASDKQDALNKEIADHKDELVALQSQFAQEQSEAAKATQAALKTAAASTASSSTSSTSNKSA
ATCC53103  SQSLSDLIARTMTVGKLSQASKDALDAVTVAKDKLAALKSEQETARQTLVSTKASLETQKSQLETLQKTASDKQDALNKEIADHKDELVALQSQFAQEQSEAAKATQAALKTAAASTASSSTSSTSNKSA
    HN001  SQSLSDLIARTMTVGKLSQASKDALDAVTVAKDKLAALKSEQETARQTLVSTKASLETQKSQLETLQKTASDKQDALNKEIADHKDELVALQSQFAQEQSEAAKATQAALKTAAASTASSSTSSTSNKSA
  LRHMDP2  SQSLSDLIARTMTVGKLSQASKDALDAVTVAKDKLAALKSEQETARQTLVSTKASLETQKSQLETLQKTASDKQDALNKEIADHKDELVALQSQFAQEQSEAAKATQAALKTAAASTASSSTSSTSNKSA
  LRHMDP3  SQSLSDLIARTMTVGKLSQASKDALDAVTVAKDKLAALKSEQETARQTLVSTKASLETQKSQLETLQKTASDKQDALNKEIADHKDELVALQSQFAQEQSEAAKATQAALKTAAASTASSSTSSTSNKSA
Consensus  SQSLSDLIARTMTVGKLSQASKDALDAVTVAKDKLAALKSEQETARQTLVSTKASLETQKSQLETLQKTASDKQDALNKEIADHKDELVALQSQFAQEQSEAAKATQAALKTAAASTASSSTSSTSNKSA

           261     270       280       290       300       310       320       330       340       350       360       370       380       390
           |--------+---------+---------+---------+---------+---------+---------+---------+---------+---------+---------+---------+---------|
    LC705  NSSVLSTGTSSTNTSSNSGASSTVISSNTASGSGSHADYSGSGNTYPWGQCTWYVKSVASWAGNGWGNGAEWGASAAAAGFTVNHTPAAGSIIVFAAGQSVGGQWTADGSYGHVAYVQSVSGDSVTITQG
 ATCC8530  NSSVLSTGTSSTNTSSNSGASSTVISSNTASGSGSHADYSGSGNTYPWGQCTWYVKSVASWAGNGWGNGAEWGASAAAAGFTVNHTPAAGSIIVFAAGQSVGGQWTADGSYGHVAYVQSVSGDSVTITQG
    R0011  NSSVLSTGTSSTNTSSNSGASSTVISSNTASGSGSHADYSGSGNTYPWGQCTWYVKSVASWAGNGWGNGAEWGASAAAAGFTVNHTPAAGSIIVFAAGQSVGGQWTADGSYGHVAYVQSVSGDSVTITQG
   LMS2-1  NSSVLSTGTSSTNTSSNSGASSTVISSNTASGSGSHADYSGSGNTYPWGQCTWYVKSVASWAGNGWGNGAEWGASAAAAGFTVNHTPAAGSIIVFAAGQSVGGQWTADGSYGHVAYVQSVSGDSVTITQG
ATCC21052  NSSVLSTGTSSTNTSSNSGASSTVISSNTASGSGSHADYSGSGNTYPWGQCTWYVKSVASWAGNGWGNGAEWGASAAAAGFTVNHTPAAGSIIVFAAGQSVGGQWTADGSYGHVAYVQSVSGDSVTITQG
       GG  NSSVLSTGTSSTNTSSNSGASSTVISSNTASGSGSHADYSGSGNTYPWGQCTWYVKSVASWAGNGWGNGAEWGASAAAAGFTVNHTPAAGSIIVFAAGQSVGGQWTADGSYGHVAYVQSVSGDSVTITQG
     E800  NSSVLSTGTSSTNTSSNSGASSTVISSNTASGSGSHADYSGSGNTYPWGQCTWYVKSVASWAGNGWGNGAEWGASAAAAGFTVNHTPAAGSIIVFAAGQSVGGQWTADGSYGHVAYVQSVSGDSVTITQG
     PEL5  NSSVLSTGTSSTNTSSNSGASSTVISSNTASGSGSHADYSGSGNTYPWGQCTWYVKSVASWAGNGWGNGAEWGASAAAAGFTVNHTPAAGSIIVFAAGQSVGGQWTADGSYGHVAYVQSVSGDSVTITQG
     PEL6  NSSVLSTGTSSTNTSSNSGASSTVISSNTASGSGSHADYSGSGNTYPWGQCTWYVKSVASWAGNGWGNGAEWGASAAAAGFTVNHTPAAGSIIVFAAGQSVGGQWTADGSYGHVAYVQSVSGDSVTITQG
ATCC53103  NSSVLSTGTSSTNTSSNSGASSTVISSNTASGSGSHADYSGSGNTYPWGQCTWYVKSVASWAGNGWGNGAEWGASAAAAGFTVNHTPAAGSIIVFAAGQSVGGQWTADGSYGHVAYVQSVSGDSVTITQG
    HN001  NSSVLSTGTSSTNTSSNSGASSTVISSNTASGSGSHADYSGSGNTYPWGQCTWYVKSVASWAGNGWGNGAEWGASAAAAGFTVNHTPAAGSIIVFAAGQSVGGQWTADGSYGHVAYVQSVSGDSVTITQG
  LRHMDP2  NSSVLSTGTSSTNTSSNSGASSTVISSNTASGSGSHADYSGSGNTYPWGQCTWYVKSVASWAGNGWGNGAEWGASAAAAGFTVNHTPAAGSIIVFAAGQSVGGQWTADGSYGHVAYVQSVSGDSVTITQG
  LRHMDP3  NSSVLSTGTSSTNTSSNSGASSTVISSNTASGSGSHADYSGSGNTYPWGQCTWYVKSVASWAGNGWGNGAEWGASAAAAGFTVNHTPAAGSIIVFAAGQSVGGQWTADGSYGHVAYVQSVSGDSVTITQG
Consensus  NSSVLSTGTSSTNTSSNSGASSTVISSNTASGSGSHADYSGSGNTYPWGQCTWYVKSVASWAGNGWGNGAEWGASAAAAGFTVNHTPAAGSIIVFAAGQSVGGQWTADGSYGHVAYVQSVSGDSVTITQG

           391     400       410    416
           |--------+---------+-----|
    LC705  GMGFSSPTGPNTQTISGASSYVYIHR
 ATCC8530  GMGFSSPTGPNTQTISGASSYVYIHR
    R0011  GMGFSSPTGPNTQTISGASSYVYIHR
   LMS2-1  GMGFSSPTGPNTQTISGASSYVYIHR
ATCC21052  GMGFSSPTGPNTQTISGASSYVYIHR
       GG  GMGFSSPTGPNTQTISGASSYVYIHR
     E800  GMGFSSPTGPNTQTISGASSYVYIHR
     PEL5  GMGFSSPTGPNTQTISGASSYVYIHR
     PEL6  GMGFSSPTGPNTQTISGASSYVYIHR
ATCC53103  GMGFSSPTGPNTQTISGASSYVYIHR
    HN001  GMGFDSPTGPNTQTISGASSYVYIHR
  LRHMDP2  GMGFDSPTGPNTQTISGASSYVYIHR
  LRHMDP3  GMGFDSPTGPNTQTISGASSYVYIHR
Consensus  GMGF.SPTGPNTQTISGASSYVYIHR
```

I

```
           1       10        20        30        40        50        60        70        80        90        100       110       120       130
           |--------+---------+---------+---------+---------+---------+---------+---------+---------+---------+---------+---------+---------|
LC705      MSFDGIFTHAMAQELNTTLSGGRVAKIQQPYENEIIITIRAGRKNHPLLLSANPQYARVQITHIPFTNPDVPATFTMTLRKYFNAATLTEIHQVQNDRVLHFEFSTRDELGDELGLRLIIEMMGRHSNIF
ATCC8530   MSFDGIFTHAMAQELNTTLSGGRVAKIQQPYENEIIITIRAGRKNHPLLLSANPQYARVQITHIPFTNPDVPATFTMTLRKYFNAATLTEIHQVQNDRVLHFEFSTRDELGDELGLRLIIEMMGRHSNIF
LMS2-1     MSFDGIFTHAMAQELNTTLSGGRVAKIQQPYENEIIITIRAGRKNHPLLLSANPQYARVQITHIPFTNPDVPATFTMTLRKYFNAATLTEIHQVQNDRVLHFEFSTRDELGDELGLRLIIEMMGRHSNIF
GG         MSFDGIFTHAMAQELNTTLSGGRVAKIQQPYENEIIITIRAGRKNHPLLLSANPQYARVQITHIPFTNPDVPATFTMTLRKYFNAATLTEIHQVQNDRVLHFEFSTRDELGDELGLRLIIEMMGRHSNIF
ATCC53103  MSFDGIFTHAMAQELNTTLSGGRVAKIQQPYENEIIITIRAGRKNHPLLLSANPQYARVQITHIPFTNPDVPATFTMTLRKYFNAATLTEIHQVQNDRVLHFEFSTRDELGDELGLRLIIEMMGRHSNIF
PEL6       MSFDGIFTHAMAQELNTTLSGGRVAKIQQPYENEIIITIRAGRKNHPLLLSANPQYARVQITHIPFTNPDVPATFTMTLRKYFNAATLTEIHQVQNDRVLHFEFSTRDELGDELGLRLIIEMMGRHSNIF
PEL5       MSFDGIFTHAMAQELNTTLSGGRVAKIQQPYENEIIITIRAGRKNHPLLLSANPQYARVQITHIPFTNPDVPATFTMTLRKYFNAATLTEIHQVQNDRVLHFEFSTRDELGDELGLRLIIEMMGRHSNIF
E800       MSFDGIFTHAMAQELNTTLSGGRVAKIQQPYENEIIITIRAGRKNHPLLLSANPQYARVQITHIPFTNPDVPATFTMTLRKYFNAATLTEIHQVQNDRVLHFEFSTRDELGDELGLRLIIEMMGRHSNIF
HN001      MSFDGIFTHAMAQELNTTLSGGRVAKIQQPYENEIIITIRAGRKNHPLLLSANPQYARVQITHIPFTNPDVPATFTMTLRKYFNAATLTEIHQVQNDRVLHFEFSTRDELGDELGLRLIIEMMGRHSNIF
ATCC21052  MSFDGIFTHSMAQELNTTLSGGRVAKIQQPYENEIIITIRAGRKNHPLLLSANPQYARVQITHIPFTNPDVPATFTMTLRKYFNAATLTEIHQVQNDRVLHFEFSTRDELGDELGLRLIIEMMGRHSNIF
R0011      MSFDGIFTHSMAQELNTTLSGGRVAKIQQPYENEIIITIRAGRKNHPLLLSANPQYARVQITHIPFTNPDVPATFTMTLRKYFNAATLTEIHQVQNDRVLHFEFSTRDELGDELGLRLIIEMMGRHSNIF
LRHMDP2    MSFDGIFTHAMAQELNTTLSGGRVAKIQQPYENEIIITIRAGRKNHPLLLSANPQYARVQITHIPFTNPDVPATFTMTLRKYFNAATLTEIHQVQNDRVLHFEFSTRDELGDELGLRLIIEMMGRHSNIF
LRHMDP3    MSFDGIFTHAMAQELNTTLSGGRVAKIQQPYENEIIITIRAGRKNHPLLLSANPQYARVQITHIPFTNPDVPATFTMTLRKYFNAATLTEIHQVQNDRVLHFEFSTRDELGDELGLRLIIEMMGRHSNIF
Consensus  MSFDGIFTH.MAQELNTTLSGGRVAKIQQPYENEIIITIRAGRKNHPLLLSANPQYARVQITHIPFTNPDVPATFTMTLRKYFNAATLTEIHQVQNDRVLHFEFSTRDELGDELGLRLIIEMMGRHSNIF

           131      140       150       160       170       180       190       200       210       220       230       240       250       260
           |--------+---------+---------+---------+---------+---------+---------+---------+---------+---------+---------+---------+---------|
LC705      LVSKRTGKIIDLIRHVSADQNRYRPLMPGAPYVEPPKQDKVDPFHDSERIYHELERQVTPSLSRATLLQQHYQGLAKDSASELALRLNQGDAGWDSFFAALATPEPTITTQGKKAVFTAIPYQSLTGEQQ
ATCC8530   LVSKRTGKIIDLIRHVSADQNRYRPLMPGAPYVEPPKQDKVDPFHDSERIYHELERQVTPSLSRATLLQQHYQGLAKDSASELALRLNQGDAGWDSFFAALATPEPTITTQGKKAVFTAIPYQSLTGEQQ
LMS2-1     LVSKRTGKIIDLIRHVSADQNRYRPLMPGAPYVEPPKQDKVDPFHDSERIYHELERQVTPSLSRATLLQQHYQGLAKDSASELALRLNQGDAGWDSFFAALATPEPTITTQGKKAVFTAIPYQSLTGEQQ
GG         LVSKRTGKIIDLIRHVSADQNRYRPLMPGAPYVEPPKQDKVDPFHDSERIYHELERQVTPSLSRAALLQQHYQGLAKDSAAELALRLNQGDAGWDSFFAALATPEPTITTQGKKAVFTAIPYQSLTGEQQ
ATCC53103  LVSKRTGKIIDLIRHVSADQNRYRPLMPGAPYVEPPKQDKVDPFHDSERIYHELERQVTPSLSRAALLQQHYQGLAKDSAAELALRLNQGDAGWDSFFAALATPEPTITTQGKKAVFTAIPYQSLTGEQQ
PEL6       LVSKRTGKIIDLIRHVSADQNRYRPLMPGAPYVEPPKQDKVDPFHDSERIYHELERQVTPSLSRAALLQQHYQGLAKDSAAELALRLNQGDAGWDSFFAALATPEPTITTQGKKAVFTAIPYQSLTGEQQ
PEL5       LVSKRTGKIIDLIRHVSADQNRYRPLMPGAPYVEPPKQDKVDPFHDSERIYHELERQVTPSLSRAALLQQHYQGLAKDSAAELALRLNQGDAGWDSFFAALATPEPTITTQGKKAVFTAIPYQSLTGEQQ
E800       LVSKRTGKIIDLIRHVSADQNRYRPLMPGAPYVEPPKQDKVDPFHDSERIYHELERQVTPSLSRAALLQQHYQGLAKDSAAELALRLNQGDAGWDSFFAALATPEPTITTQGKKAVFTAIPYQSLTGEQQ
HN001      LVSKRTGKIIDLIRHVSADQNRYRPLMPGAPYVEPPKQDKVDPFHDSERIYHELERQVTPSLSRAALLQQHYQGLAKDSAAELALRLNQGDAGWDSFFAALATPEPTITTQGKKAVFTAIPYQSLTGEQQ
ATCC21052  LVSKRTGKIIDLIRHVSADQNRYRPLMPGAPYVEPPKQDKVDPFHDSERIYHELERQVTPSLSRATLLQQHYQGLAKDSAAELALRLNQGDAGWDSFFAALATPEPTITTQGKKAVFTAIPYQSLTGEQQ
R0011      LVSKRTGKIIDLIRHVSADQNRYRPLMPGAPYVEPPKQDKVDPFHDSERIYHELERQVTPSLSRATLLQQHYQGLAKDSAAELALRLNQGDAGWDSFFAALATPEPTITTQGKKAVFTAIPYQSLTGEQQ
LRHMDP2    LVSKRTGKIIDLIRHVSADQNRYRPLMPGAPYVEPPKQDKVDPFHDSERIYHELERQVTPSLSRAALLQQRYQGLAKDSAAELALRLNQGDAGWDSFFVALATPEPTITTQGKKAVFTAIPYQSLTGEQQ
LRHMDP3    LVSKRTGKIIDLIRHVSADQNRYRPLMPGAPYVEPPKQDKVDPFHDSERIYHELERQVTPSLSRAALLQQRYQGLAKDSAAELALRLNQGDAGWDSFFVALATPEPTITTQGKKAVFTAIPYQSLTGEQQ
Consensus  LVSKRTGKIIDLIRHVSADQNRYRPLMPGAPYVEPPKQDKVDPFHDSERIYHELERQVTPSLSRA.LLQQ.YQGLAKDSA.ELALRLNQGDAGWDSFF.ALATPEPTITTQGKKAVFTAIPYQSLTGEQQ

           261      270       280       290       300       310       320       330       340       350       360       370       380       390
           |--------+---------+---------+---------+---------+---------+---------+---------+---------+---------+---------+---------+---------|
LC705      HFSTLSAMLDAYYAQKAEHDRVLQQGGNLIHVIKNVIDKDRKKQRKLKRTLEETEKADDYRIRGEILTTYLSQVKRGMTSIELPNFYADNEPIKITLSNQLTPSRNAQKYFAKYTKLRNAVAHVHQQMQE
ATCC8530   HFSTLSAMLDAYYAQKAEHDRVLQQGGNLIHVIKNVIDKDRKKQRKLKRTLEETEKADDYRIRGEILTTYLSQVKRGMTSIELPNFYADNEPIKITLSNQLTPSRNAQKYFAKYTKLRNAVAHVHQQMQE
LMS2-1     HFSTLSAMLDAYYAQKAEHDRVLQQGGNLIHVIKNVIDKDRKKQRKLKRTLEETEKADDYRIRGEILTTYLSQVKRGMTSIELPNFYADNEPIKITLSNQLTPSRNAQKYFAKYTKLRNAVAHVHQQMQE
GG         HFPTLSAMLDAYYAQKAEHDRVLQQGGNLIHVIKNVIDKDRKKQRKLKRTLEETEKADDYRIRGEILTTYLSQVKRGMTSIELPNFYADNEPIKITLSNQLTPSRNAQKYFAKYTKLRNAVAHVHQQMQE
ATCC53103  HFPTLSAMLDAYYAQKAEHDRVLQQGGNLIHVIKNVIDKDRKKQRKLKRTLEETEKADDYRIRGEILTTYLSQVKRGMTSIELPNFYADNEPIKITLSNQLTPSRNAQKYFAKYTKLRNAVAHVHQQMQE
PEL6       HFPTLSAMLDAYYAQKAEHDRVLQQGGNLIHVIKNVIDKDRKKQRKLKRTLEETEKADDYRIRGEILTTYLSQVKRGMTSIELPNFYADNEPIKITLSNQLTPSRNAQKYFAKYTKLRNAVAHVHQQMQE
PEL5       HFPTLSAMLDAYYAQKAEHDRVLQQGGNLIHVIKNVIDKDRKKQRKLKRTLEETEKADDYRIRGEILTTYLSQVKRGMTSIELPNFYADNEPIKITLSNQLTPSRNAQKYFAKYTKLRNAVAHVHQQMQE
E800       HFPTLSAMLDAYYAQKAEHDRVLQQGGNLIHVIKNVIDKDRKKQRKLKRTLEETEKADDYRIRGEILTTYLSQVKRGMTSIELPNFYADNEPIKITLSNQLTPSRNAQKYFAKYTKLRNAVAHVHQQMQE
HN001      HFPTLSAMLDAYYAQKAEHDRVLQQGGNLIHVIKNVIDKDRKKQRKLKRTLEETEKADDYRIRGEILTTYLSQVKRGMTSIELPNFYADNEPIKITLSNQLTPSRNAQKYFAKYTKLRNAVAHVHQQMQE
ATCC21052  HFPTLSAMLDAYYAQKAEHDRVLQQGGNLIHVIKNVIDKDRKKQRKLKRTLEETEKADDYRIRGEILTTYLSQVKRGMTSIELPNFYADNEPIKITLSNQLTPSRNAQKYFAKYTKLRNAVAHVHQQMQE
R0011      HFPTLSAMLDAYYAQKAEHDRVLQQGGNLIHVIKNVIDKDRKKQRKLKRTLEETEKADDYRIRGEILTTYLSQVKRGMTSIELPNFYADSEPIKITLSNQLTPSRNAQKYFAKYTKLRNAVAHVHQQMQE
LRHMDP2    HFPTLSAMLDAYYAQKAEHDRVLQQGGNLIHVIKNVIDKDRKKQRKLKRTLEETEKADDYRIRGEILTTYLSQVKRGMTSIELPNFYADNEPIKITLSNQLTPSRNAQKYFAKYTKLRNAVAHVHQQMKE
LRHMDP3    HFPTLSAMLDAYYAQKAEHDRVLQQGGNLIHVIKNVIDKDRKKQRKLKRTLEETEKADDYRIRGEILTTYLSQVKRGMTSIELPNFYADNEPIKITLSNQLTPSRNAQKYFAKYTKLRNAVAHVHQQMKE
Consensus  HF.TLSAMLDAYYAQKAEHDRVLQQGGNLIHVIKNVIDKDRKKQRKLKRTLEETEKADDYRIRGEILTTYLSQVKRGMTSIELPNFYAD.EPIKITLSNQLTPSRNAQKYFAKYTKLRNAVAHVHQQM.E

           391      400       410       420       430       440       450       460       470       480       490       500       510       520
           |--------+---------+---------+---------+---------+---------+---------+---------+---------+---------+---------+---------+---------|
LC705      NQEELDYLEGIMAQIDVASPKDLVDIRLELQQQGYLRKQKSGKKGNKRQKVSKPDQFYASDGTKIWVGKNNLQNDQLTLHTAKKTDIWLHVKDIPGSHVIIDSSDPSEKTLLEAAELAAYFSKARDSANV
ATCC8530   NQEELDYLEGIMAQIDVASPKDLVDIRLELQQQGYLRKQKSGKKGNKRQKVSKPDQFYASDGTKIWVGKNNLQNDQLTLHTAKKTDIWLHVKDIPGSHVIIDSSDPSEKTLLEAAELAAYFSKARDSANV
LMS2-1     NQEELDYLEGIMAQIDVASPKDLVDIRLELQQQGYLRKQKSGKKGNKRQKVSKPDQFYASDGTKIWVGKNNLQNDQLTLHTAKKTDIWLHVKDIPGSHVIIDSSDPSEKTLLEAAELAAYFSKARDSANV
GG         NQEELDYLEGIMAQIDVASPKDLVDIRLELQQQGYLRKQKSGKKGNKRQKVSKPDQFYASDGTKIWVGKNNLQNDQLTLHTAKKTDIWLHVKDIPGSHVIIDSSDPSEKTLLEAAKLAAYFSKARDSANV
ATCC53103  NQEELDYLEGIMAQIDVASPKDLVDIRLELQQQGYLRKQKSGKKGNKRQKVSKPDQFYASDGTKIWVGKNNLQNDQLTLHTAKKTDIWLHVKDIPGSHVIIDSSDPSEKTLLEAAKLAAYFSKARDSANV
PEL6       NQEELDYLEGIMAQIDVASPKDLVDIRLELQQQGYLRKQKSGKKGNKRQKVSKPDQFYASDGTKIWVGKNNLQNDQLTLHTAKKTDIWLHVKDIPGSHVIIDSSDPSEKTLLEAAKLAAYFSKARDSANV
PEL5       NQEELDYLEGIMAQIDVASPKDLVDIRLELQQQGYLRKQKSGKKGNKRQKVSKPDQFYASDGTKIWVGKNNLQNDQLTLHTAKKTDIWLHVKDIPGSHVIIDSSDPSEKTLLEAAKLAAYFSKARDSANV
E800       NQEELDYLEGIMAQIDVASPKDLVDIRLELQQQGYLRKQKSGKKGNKRQKVSKPDQFYASDGTKIWVGKNNLQNDQLTLHTAKKTDIWLHVKDIPGSHVIIDSSDPSEKTLLEAAKLAAYFSKARDSANV
HN001      NQEELDYLEGIMAQIDVASPKDLVDIRLELQQQGYLRKQKSGKKGNKRQKVSKPDQFYASDGTKIWVGKNNLQNDQLTLHTAKKTDIWLHVKDIPGSHVIIDSSDPSEKTLLEAAKLAAYFSKARDSANV
ATCC21052  NQEELDYLEGIMAQIDVASPKDLVDIRLELQQQGYLRKQKSGKKGNKRQKVSKPDQFYASDGTKIWVGKNNLQNDQLTLHTAKKTDIWLHVKDIPGSHVIIDSSDPSEKTLLEAAKLAAYFSKARDSANV
R0011      NQEELDYLEGIMAQIDVASPKDLVDIRLELQQQGYLRKQKSGKKGNKRQKVSKPDQFYASDGTKIWVGKNNLQNDQLTLHTAKKTDIWLHVKDIPGSHVIIDSSDPSEKTLLEAAKLAAYFSKARDSANV
LRHMDP2    NQEELDYLEGIMAQIDVASPKDLVDIRLELQQQGYLRKQKSGKKGNKRQKVSKPDQFYASDGTKIWVGKNNLQNDQLTLHTAKKTDIWLHVKDIPGSHVIIDSSDPSEKTLLEAAKLAAYFSKARDSANV
LRHMDP3    NQEELDYLEGIMAQIDVASPKDLVDIRLELQQQGYLRKQKSGKKGNKRQKVSKPDQFYASDGTKIWVGKNNLQNDQLTLHTAKKTDIWLHVKDIPGSHVIIDSSDPSEKTLLEAAKLAAYFSKARDSANV
Consensus  NQEELDYLEGIMAQIDVASPKDLVDIRLELQQQGYLRKQKSGKKGNKRQKVSKPDQFYASDGTKIWVGKNNLQNDQLTLHTAKKTDIWLHVKDIPGSHVIIDSSDPSEKTLLEAA.LAAYFSKARDSANV

           521      530       540       550       560    567
           |--------+---------+---------+---------+------|
LC705      PVDWIEVKKIRKPNGAKPGFVIYEGQKTVSVTPDADLVAKLRNPPTK
ATCC8530   PVDWIEVKKIRKPNGAKPGFVIYEGQKTVSVTPDADLVAKLRNPPTK
LMS2-1     PVDWIEVKKIRKPNGAKPGFVIYEGQKTVSVTPDADLVAKLRNPPTK
GG         PVDWIEVKKIRKPNGAKPGFVIYEGQKTVSVTPDADLVAKLRNPPTK
ATCC53103  PVDWIEVKKIRKPNGAKPGFVIYEGQKTVSVTPDADLVAKLRNPPTK
PEL6       PVDWIEVKKIRKPNGAKPGFVIYEGQKTVSVTPDADLVAKLRNPPTK
PEL5       PVDWIEVKKIRKPNGAKPGFVIYEGQKTVSVTPDADLVAKLRNPPTK
E800       PVDWIEVKKIRKPNGAKPGFVIYEGQKTVSVTPDADLVAKLRNPPTK
HN001      PVDWIEVKKIRKPNGAKPGFVIYEGQKTVSVTPDADLVAKLRNPPTK
ATCC21052  PVDWIEVKKIRKPNGAKPGFVIYEGQKTVSVTPDADLVAKLRNPPTK
R0011      PVDWIEVKKIRKPNGAKPGFVIYEGQKTVSVTPDADLVAKLRNPPTK
LRHMDP2    PVDWIEVKKIRKPNGAKPGFVIYEGQKTVSVTPDVDLVAKLRNPPTK
LRHMDP3    PVDWIEVKKIRKPNGAKPGFVIYEGQKTVSVTPDVDLVAKLRNPPTK
Consensus  PVDWIEVKKIRKPNGAKPGFVIYEGQKTVSVTPD.DLVAKLRNPPTK
```
